# Strong Coupling between Localized Surface Plasmons and Molecules by Coupled Cluster Theory

**DOI:** 10.1021/acs.nanolett.1c02162

**Published:** 2021-07-20

**Authors:** Jacopo Fregoni, Tor S. Haugland, Silvio Pipolo, Tommaso Giovannini, Henrik Koch, Stefano Corni

**Affiliations:** † Dipartimento di Scienze Chimiche, University of Padova, I-35131 Padova, Italy; ‡ Institute of Nanosciences, Consiglio Nazionale delle Ricerche CNR-Nano, I-41125 Modena, Italy; § Department of Chemistry, Norwegian University of Science and Technology, 7491 Trondheim, Norway; ∥ UCCS Unité de Catalyse et Chimie du Solide, Université de Lille, Université d’Artois UMR 8181, F-59000, Lille, France; ⊥ Scuola Normale Superiore, I-56126, Pisa, Italy; # Dipartimento di Scienze Chimiche and Padua Quantum Technologies Research Center, University of Padova, I-35131 Padova, Italy

**Keywords:** Plexcitons, Polaritonic Chemistry, Cavity-QED, Quantum Nanoparticles, Quantum Chemistry, Nanoplasmonics, Quantum coupling

## Abstract

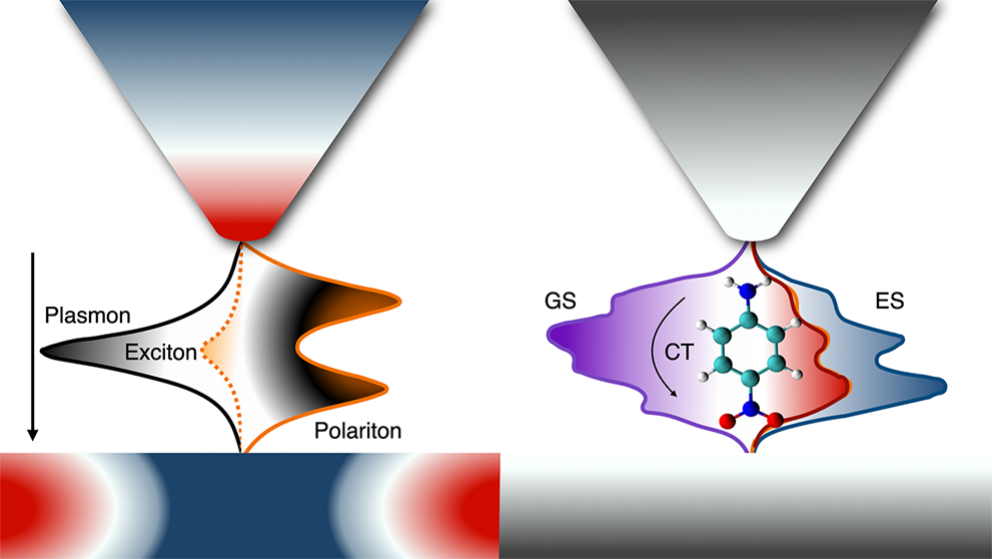

Plasmonic nanocavities enable the confinement of molecules and electromagnetic fields within nanometric volumes. As a consequence, the molecules experience a remarkably strong interaction with the electromagnetic field to such an extent that the quantum states of the system become hybrids between light and matter: polaritons. Here, we present a nonperturbative method to simulate the emerging properties of such polaritons: it combines a high-level quantum chemical description of the molecule with a quantized description of the localized surface plasmons in the nanocavity. We apply the method to molecules of realistic complexity in a typical plasmonic nanocavity, featuring also a subnanometric asperity (picocavity). Our results disclose the effects of the mutual polarization and correlation of plasmons and molecular excitations, disregarded so far. They also quantify to what extent the molecular charge density can be manipulated by nanocavities and stand as benchmarks to guide the development of methods for molecular polaritonics.

Strong coupling between molecules and quantum plasmons^1^ in nanocavities^2^ leads to the formation of hybrid plasmon–molecule states: polaritons, or more specifically, plexcitons.^3^ These new states manifest distinct features compared to the original states,^4−7^ potentially resulting in modified chemical/photochemical reactivity^8−10^ and relaxation dynamics,^11,12^ along with other coherent processes.^13,14^ Modeling accurately the molecules, nanostructures and their coupling is of the utmost importance to support the experimental advances. Coupling descriptions proper for resonant optical cavities, such as using the molecular dipole only,^15,16^ should be amended^17−19^ when the coupling affects the molecule on a submolecular level.^18,20,21^

There are currently no approaches that treat the coupled plasmon–molecule system nonperturbatively, that is, that include the relaxation of the molecular electronic density upon polariton formation together with plasmon–molecule correlation. In this work we extend the QED-CC method,^22,23^ already applied to resonant optical cavities, to realistic nanoplasmonic cavities. We quantize the plasmonic excitations starting from the classical dielectric description of nanostructures with arbitrary shapes. Each quantized plasmonic mode is associated with a surface charge density (discretized into point charges), analogous of a transition charge density in a molecular system. A similar quantization approach for plasmons in Drude metals, derived from macroscopic QED,^19,24,25^ was adopted in ref (17). The quantization framework we are presenting accounts for the geometrical features of the nanoparticles setups without disregarding the molecular complexity. Indeed, coupled cluster (singles and doubles excitations) is recognized as a highly accurate method in quantum chemistry:^26^ we extend it to include self-consistent and correlated molecule–plasmon hybridization. With this method, we compute the interaction between a nanocavity realized after ref (18) and two realistic molecules: porphyrin and para-nitroaniline (PNA). For porphyrin, we highlight the role of the geometrical features of the system and the role of electron–plasmon self-consistency and correlation in polaritons, beyond the standard Jaynes-Cumming picture.^27^ We use PNA to quantify the same effects, already predicted on the ground and excited states electron densities^22^ for a resonant cavity, in the case of a nanocavity.

## Results and Discussion

### Theory

The first subsystem we focus on is the nanostructure. We start from a classical dielectric perspective, namely computing the plasmon modes from a continuous medium model. As such, it is not an atomistic description and does not include dielectric nonlocal effects, electron spillout, or chemical bonds. However, the description we adopt is surprisingly accurate:^28^ it reproduces the inhomogeneous electric field due to atomistic irregularities in the nanostructures^29^ even at subnanometric level.^30^ Our goal is to describe the nanostructure at a quantum level as a material system, which differs from other approaches that quantize the electromagnetic field in the presence of dielectric media.^24,31^ By building on polarizable continuum model applied to nanoparticles (PCM-NP) approach,^32^ we shall achieve quantization (Q-PCM-NP) in the quasi-electrostatic framework, where the retardation effects are disregarded.^33^ Similar quantization frameworks for the plasmonic modes were used in different contexts not involving a molecule^34^ or using a model description of the molecule.^35^ To classically compute the nanoparticle dielectric response properties, we solve Maxwell’s equations for a continuous, frequency-dependent dielectric (nanostructure) under an external electromagnetic perturbation. In the quasi-static limit, the nanoparticle experiences an electrostatic potential at a given frequency, **V**(ω), which induces polarization charges **q**(ω) on the nanoparticle surface. The equation defining such response charges is
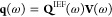
1where **Q**^IEF^(ω) is the frequency-dependent response to the external perturbation **V**(ω). We refer to IEF as to the specific integral equation formalism^36^ formulation of the PCM problem. In the present work, **V**(ω) is the potential produced by the molecular transition density. The frequency-dependent linear charge density response function of the nanostructure is taken in a diagonal form^37^
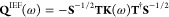
2
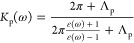
3where **K** is the diagonal linear-charge-response-matrix derived from eigenvalues Λ_p_ of the appropriate IEF matrix,^37^ ε(ω) is the frequency-dependent dielectric function, and **S** is the matrix storing the electrostatic potential between discrete points of the dielectric. This is the starting point of the Q-PCM-NP quantization procedure.

### Quantization of the Plasmonic Modes: Q-PCM-NP

We assume that the metal nanoparticle can be characterized by a Drude-Lorentz dielectric function
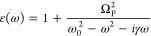
4where Ω_P_^2^ is the squared plasma frequency of the bulk metal, ω_0_ is the natural frequency of the bound oscillators (Lorentz model), and γ is the damping rate. By defining  (neglecting the second-order term in γ), we retrieve (see Supporting Information) the full form of the response function **Q**^IEF^(ω) from eqs 3 and 4
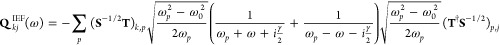
5

Each matrix element of **Q**^IEF^(ω) is evaluated on representative points of the tesserae, *k* and *j*. In eq 5, we recognize the spectral form of a linear response function,^38^**Q**^quant^(ω). Our goal now is to identify the quantities characterizing the excited states of the nanostructure (that is, plasmons) relevant to devise the coupling with the molecule. For the quantum description of the nanostructure, the response function **Q**^quant^(ω) is formally written in terms of the surface charge operator *q̂*.^39^ We conveniently write **Q**^quant^(ω) matrix elements in its spectral representation^40^ as
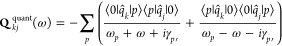
6

**Figure 1:**
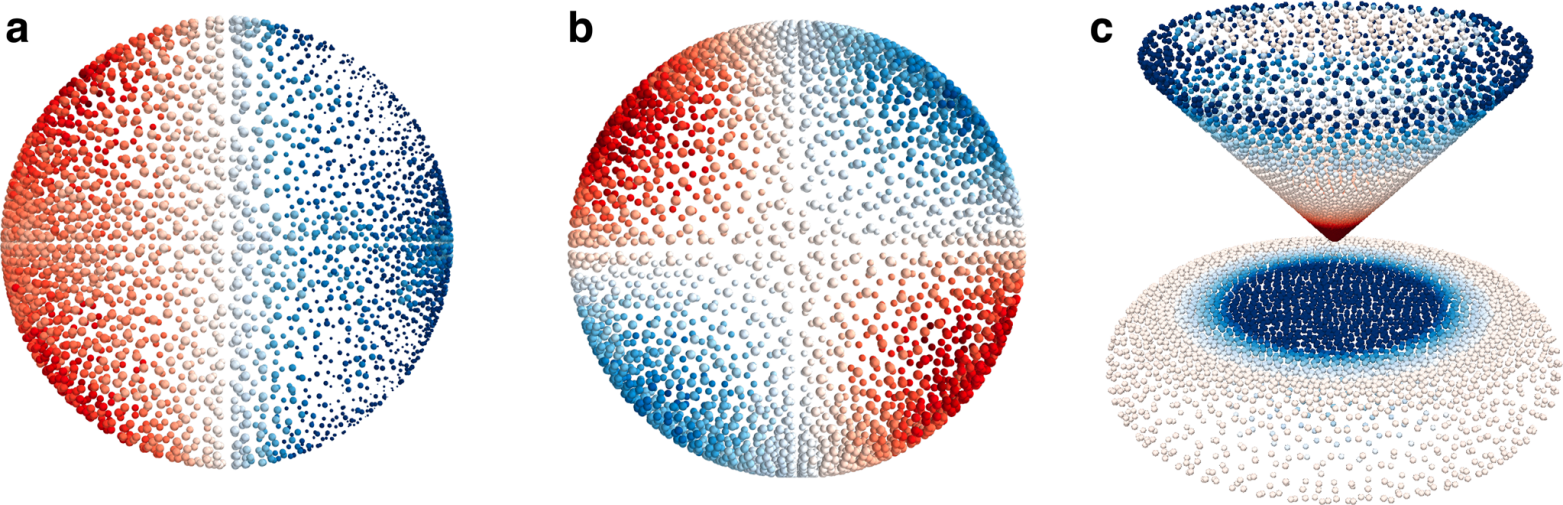
Examples of plasmonic modes computed by Q-PCM-NP. (a) Example of plasmonic dipolar mode for a 10 nm sphere. The *p* index associated with each set of charges does not take into account the 2*l* + 1 degeneracy for the modes of the sphere. Hence, the dipolar modes are *p* = 2 to *p* = 4. (b) Example of a quadrupolar mode for the same sphere. Similar as for the dipolar case, the quadrupolar modes are 2*l* + 1 degenerate, hence they are from *p* = 5 to *p* = 9 and so on. (c) Relevant plasmonic mode for a nanotip on a support. The mode is characterized by a strong charge density concentrated on the tip as in ref (18). The dimensions of the system are about 10 × 10 nm (see Figure S1b)

By comparing eq 5 and eq 6, we verify that ω_*p*_ represents the excitation frequencies of the plasmonic system. γ_*p*_′ = γ/2 for each plasmon state *p* is a decay rate, while we can identify ⟨0|*q̂*_*k*_|*p*⟩ with
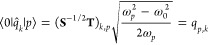
7The label *q*_*p*,*k*_ represents the transition charge sitting on the *k*th tessera associated with the mode *p* and they are the analogous of transition densities in molecules. Expressed differently, the ensemble of the *q*_*p*,*k*_ set of charges represents the normal mode of the plasmonic system at frequency ω_*p*_ (see Figure 1a–c as an example).

We are now in the position to write the quantum plasmonic Hamiltonian as
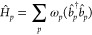
8where ω_*p*_ is the *p*-mode frequency and *b̂*_*p*_^†^, *b̂*_*p*_ are the corresponding bosonic creation and annihilation operators.^17,25^ Assuming the localized surface plasmons to be bosons is not a result of the present derivation (at the linear response level, a Fermionic or bosonic excitation would yield the same **Q**^quant^(ω)). It is rather based on the general behavior of plasmons in extended systems.^1^ In the Supporting Information, we report numerical checks of the present approach against analytical results for a point-dipole molecule interacting with a Drude metal nanosphere.^41^ We can now write the general quantized plasmon–molecule Hamiltonian as
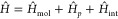
9where *Ĥ*_mol_ is the standard molecular Hamiltonian (which we reduce to the electronic Hamiltonian). Making use of the response charges for the quantized plasmon, the interaction term *Ĥ*_int_ reads^39^
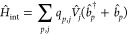
10where *V̂*_*j*_ is the molecular electrostatic potential operator, evaluated at each tessera representative point *j*. Our formulation of *Ĥ*_int_ presents two major advances with respect to simplified models (see Supporting Information for the formal comparison): we obtain an intuitive representation of the inhomogeneous electromagnetic field in terms of polarization charges and the interaction is general on the molecular side, meaning that it can be interfaced to advanced electronic structure models. Through this interface, our method can describe phenomena occurring in both the weak and strong coupling regime.^42,43^ Radiative corrections to the quasi-static description, related to radiation reaction,^44^ can also be included in this model, at the quantum level. The plasmon radiative decay rate can be calculated from the transition dipole moment associated with the charges *q*_*p*,*j*_ and it can then be added to the nonradiative lifetime γ_*p*_. For the systems treated in the numerical section, this correction is negligible. An estimate of the molecule–plasmon coupling *g*_*n*_ between the plasmonic mode *p* and the molecular electronic transition from the ground state *S*_0_ to the excited state *S*_*n*_ is given by
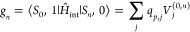
11Here 0 and 1 are the occupation numbers of the plasmonic mode *p*. The coupling *g*_*n*_ is the central quantity in simplified approaches to strong coupling, such as the Jaynes-Cummings model (JC).

### Extending QED Coupled Cluster (QED-CC) to Plasmon–Molecule Systems

Determining the eigenfunctions of the plasmon–molecule Hamiltonian in eq 9 requires an accurate description of the molecule. In this section, we outline the extension of the QED-CC treatment (already applied to resonant cavities^22,23^) to the case of plasmons. The detailed algorithm is available in the QED-CC dedicated section of the Supporting Information. The simplest description of the molecule without plasmon interactions is a single Slater determinant, |HF⟩ (Hartree–Fock). To include electron correlation we rely on coupled cluster theory.^26^ More explicitly, we apply the exponential of the cluster operator to the |HF⟩ determinant
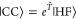
12

The cluster operator *T̂* is expressed as
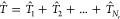
13where *T*_1_ are linear combinations of single excitations, *T*_2_ are linear combinations of double excitations and so on. Inspired by the formal similarities of the interaction in molecule–plasmon and molecule–photon systems, we extend the newly developed quantum electrodynamics coupled cluster theory (QED-CC)^22^ from photons to plasmons. Here the plasmon–molecule interaction is described nonperturbatively, allowing also the ground state to couple to the electromagnetic field.^23^ In QED-CC, the cluster operator *T̂* also includes the bosonic creation and annihilation operators *b̂*_*p*_^†^, *b̂*_*p*_ of the quantized plasmon mode *p*
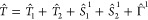
14

Here we identify three contributions: electronic (*T̂*_1_, *T̂*_2_), plasmonic (Γ̂^1^) and mixed plasmon–molecule excitations (*Ŝ*_1_^1^, *Ŝ*_2_^1^). By the effect of such excitations, QED-CCSD-1 (SD denotes single and double excitation of the electronic subspace, whereas 1 is the excitation order of the plasmonic modes) includes the correlation induced on the electronic states by the plasmonic transition. We note that, in the limit where the interaction *ĝ* → 0, CCSD and QED-CCSD-1 are equivalent.

### Calculations with Molecules

#### Free-Base Porphyrin

**Figure 2:**
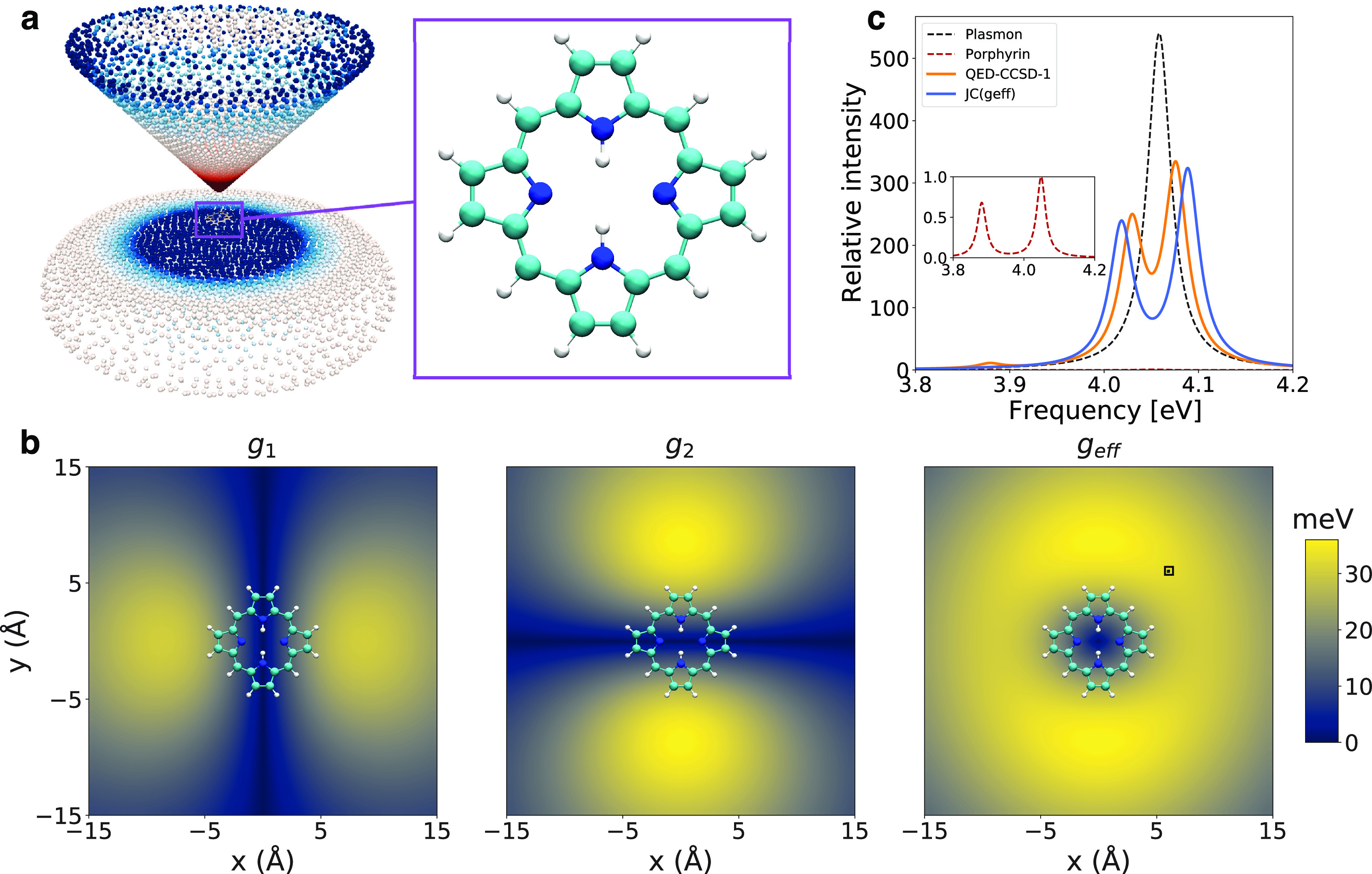
Interaction between the free-base porphyrin and a nanotip. (a) Setup for porphyrin interacting with a plasmonic nanotip used in panels b and c, where the nanotip has transition energy ω = 4.06 eV. (b) Map of the plasmon–molecule coupling *g* for two excitations in porphyrin (4.05 eV, 4.07 eV) and the effective coupling . Axis *x* and axis *y* indicate the displacement of the nanotip from the center of the molecule. (c) Oscillator strengths (intensity) for porphyrin displaced 6 Å along *x* and 6 Å along *y* (see black square in b)). Intensities are relative to porphyrin’s strongest transition. The inset shows the same plot with only porphyrin. JC(g_eff_) refers to a two state JC model where the coupling and intensities refer to g_eff_.

In this section, we exploit Q-PCM-NP coupled to QED-CCSD-1 and EOM-CCSD to simulate plasmon–molecule interactions. Here we consider a free-base porphyrin coupled to the plasmonic nanotip, as sketched in Figure 2a. A similar setup was adopted in refs (17 and 18). According to eq 11, we define the couplings between the ground state |*S*_0_, 1⟩ of the free-base porphyrin and the |*S*_1_, 0⟩, |*S*_2_, 0⟩ states as *g*_1_ and *g*_2_ respectively. In Figure 2b, we show the associated interaction maps, where the couplings *g*_1_ and *g*_2_ are displayed as a function of the molecule–nanotip displacement. The two quasi-degenerate dipolar transitions of free-base porphyrin at 4.05 eV (*S*_1_) and 4.07 eV (*S*_2_) have different symmetries. Depending on the tip displacement, the interaction element *g* varies between 0 meV at the center to 36 meV at 6 Å displacement. By displacing the tip accordingly, a polariton is created with either of the two states. When combining the two quasi-degenerate transitions, , a doughnut-like shape of the transition density is obtained, in agreement with previous results on zinc phtalocyanine.^17^ We stress that this doughnut-like shape is not observable without including the geometry of the nanostructure. In Figure 2c, we show the absorption spectrum of the relaxed tip–molecule complex (QED-CCSD-1) and compare it with the polaritons obtained by the Jaynes-Cummings model (see the Supporting Information for details). The point at which we perform the calculations is highlighted as the black square in panel *g*_eff_ of Figure 2b. In QED-CCSD-1, the light–matter system is allowed to correlate both the ground and the excited states, guaranteeing accurate results with respect to the truncated basis. Even more, the interactions between all singly and doubly excited determinants with and without plasmons are included. Conversely, the JC case is treated by correlating the electronic states only, hence neglecting the mixed molecule–plasmon correlation contributions. As a further difference between JC and QED-CCSD-1, the interaction in the former is a simple norm of the *g*_1_ and *g*_2_ interactions, and it does not allow for the *S*_1_ and *S*_2_ states to interact through the plasmon. For QED-CCSD-1, the presence of the polaritonic correlation and several electronic states reveals that the plasmon contribution is distributed over other electronic states displayed as the small peak at 3.88 eV in Figure 2c. At the same time, the Rabi splitting between the two main polaritonic peaks is reduced (46 meV) with respect to JC (70 meV). While the small peak is due to the inclusion of different states, the difference in the Rabi splitting is due to the inclusion of the mixed plasmon–molecule correlation. To corroborate this view, we compare in the Supporting Information an extended JC model using three states with the QED-CCSD-1. Although the description improves with the extended JC model, it still misses the intensities and the positions of the polaritonic peaks, confirming the role of the mixed plasmon–molecule correlation in the description.

#### Para-nitroaniline

Para-nitroaniline (PNA) presents an intense absorption peak in the UV range. We hence expect large Rabi splittings when the PNA is appropriately aligned with the electromagnetic field of the nanotip. The transition density associated with the bright transition of PNA at ∼4.5 eV is parallel to the long axis of the molecule, making it an excellent candidate for single-molecule strong coupling.

**Figure 3:**
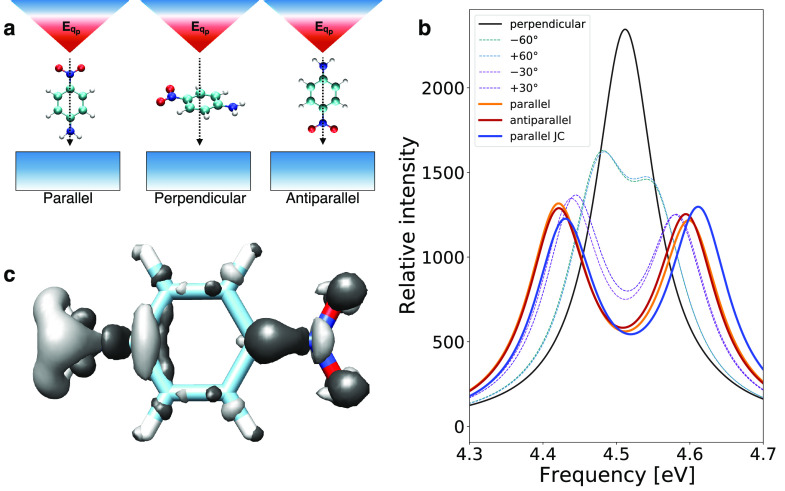
Quantum coupling between a PNA molecule and a nanotip. (a) Sketch of the molecule interacting with the plasmonic nanotip. The parallel orientations identify the maximum plasmon–molecule coupling condition, whereas the perpendicular one corresponds to the minimum coupling. (b) PNA oscillator strength (intensity) at different orientations inside a plasmonic nanotip-cavity. Each calculation is performed at different molecular orientations around the nuclear center of charge. The plasmon is resonant with the bright molecular transition (4.52 eV). The comparison between the realistic structure of the molecule (QED-CCSD) and the two-level JC model is shown by the full colored lines. (c) Plasmon-induced difference in PNA’s ground-state density, when PNA is parallel with the plasmonic mode of frequency ω = 4.52 eV. Black and white isosurfaces indicate increased and reduced density (±10^*–*3^*e*^–^/*a*_0_^3^).

In Figure 3, we investigate the coupling of the PNA molecule with the nanotip. We identify the maximum (parallel) and the minimum (perpendicular) coupling conditions, as sketched in Figure 3a, and plot the Rabi splitting of the plasmon–molecule coupled system in Figure 3b. When the molecular transition dipole is perpendicular to the plasmon mode, there is little-to-no effect on the spectrum. Instead, when the molecule is gradually rotated to be parallel to the mode, the Rabi splitting increases until the maximum of 179 meV (parallel). By gradually rotating the molecule in the opposite direction (antiparallel) we observe a slightly smaller splitting (175 meV). The 4 meV difference between the parallel and antiparallel orientations is a signature of the realistic molecular description. Indeed, a dipolar interaction would result in a symmetric spectrum with a Rabi splitting independent of the parallel/antiparallel orientation. In addition, we observe a red-shift of the polaritonic peaks when including the mixed plasmon–molecule correlation energy via QED-CCSD-1.

**Figure 4:**
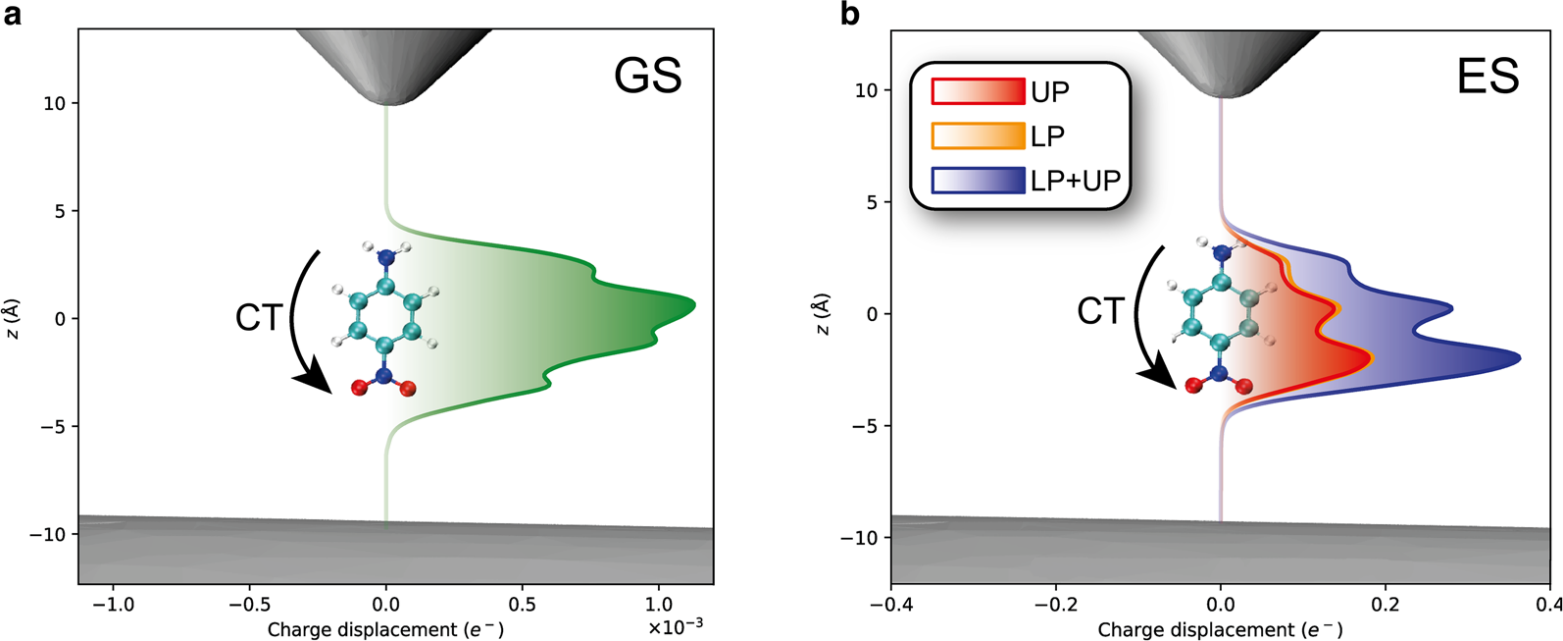
Charge displacement analysis of PNA. The molecule is placed parallel with respect to the plasmonic nanotip. The direction of charge transfer (CT) is along the arrow. (a) Plasmon-induced density difference in the ground state. (b) Charge transfer excitation around 4.5 eV. The red and orange areas are upper (UP) and lower (LP) polaritons, respectively. The blue curve is the sum of red and orange.

The Jaynes-Cummings model fails at describing ground-state properties, as no change in the molecular ground state is predicted by the model. By instead using QED-CCSD-1, we show in Figure 3c how the ground-state electronic density changes due to the molecule–plasmon interaction. Since the coupling in the present case is smaller than the one considered in ref (22) the charge localization effects as discussed in the same reference are reduced. We quantify this effect in Figure 4a. We note the opposite trend to what was described in ref (22), namely that here the plasmon induces a small charge transfer from the donor to the acceptor. This inversion is a signature that the molecules interact differently with plasmonic nanotips compared to optical cavities, whereas the Jaynes-Cummings model treats them equivalently. The charge transfer properties of the polariton states are also substantially changed, reminiscent of what is shown in ref (22). However, in the present case the sum of the polaritons’ charge transfer adds up to the charge transfer of the singlet state without the plasmons, as displayed in Figure 4b. This again highlights that interactions with nanoparticle plasmons and optical cavities are not equivalent. The plasmon-induced changes in the electronic density also imply a modification of other ground-state properties as well as the potential energy surfaces. One example is the minor induced change of the dipole moment (− 0.03 D). One important limitation in this ground-state study is the inclusion of only a single plasmon mode: higher energy modes are expected to contribute to the ground-state coupling and enhance the minor effects seen here, as the ground-state correlation is not a resonant property.^23^

## Conclusions

In this work, we have introduced a new methodology, based on PCM-NP^45,46^ and QED-CC,^22^ to treat the quantum coupling between nanoparticles of arbitrary shapes and molecules. We compared our description of polaritons in free-base porphyrin with the simplified Jaynes-Cummings model, highlighting the role of the mixed molecule–plasmon correlation. Finally, we have analyzed the effects on the ground state of PNA in a realistic plasmonic nanocavity setup, showing that the modification of ground states density previously investigated by QED-CC is still present. Our representation of quantum plasmonic modes as apparent surface charges, in synergy with coupled cluster, provides a simple-yet-accurate interface to tackle mixed nanoparticle–molecules systems. In conclusion, we believe our method provides solid ground to support and guide experiments investigating and manipulating the chemical properties at the submolecular level. Accurate calculations of plexciton-affected reaction-barriers are already possible, as well as exploring effects related to chiral nanotips. The method is also prone to various extensions on the nanostructure side: the inclusion of multiple EM modes or the extension to classical atomistic models.^47−49^ Finally, the model may assess the properties of molecules within plasmonic nanocavities in turn hosted by optical cavities, a setup whose experimental exploration has been recently started.^50^

## Supporting Information Available

Details of the theoretical and computational specifics for Q-PCM-NP and QED-CC and further benchmarks and supplementary results; computational details for the reproducibility of the calculations and the codes availability (PDF)

## References

[ref1] Tame M.; Mcenery K. R.; Ozdemir S.; Lee J.; Maier S.; Kim M. Quantum plasmonics. Nat. Phys. 2013, 9, 329–340. 10.1038/nphys2615.

[ref2] Hugall J. T.; Singh A.; van Hulst N. F. Plasmonic Cavity Coupling. ACS Photonics 2018, 5, 43–53. 10.1021/acsphotonics.7b01139.

[ref3] Fofang N. T.; Grady N. K.; Fan Z.; Govorov A. O.; Halas N. J. Plexciton Dynamics: Exciton Plasmon Coupling in a J Aggregate Au Nanoshell Complex Provides a Mechanism for Nonlinearity. Nano Lett. 2011, 11, 1556–1560. 10.1021/nl104352j. 21417362

[ref4] Li J.-F.; Li C.-Y.; Aroca R. F. Plasmon-enhanced fluorescence spectroscopy. Chem. Soc. Rev. 2017, 46, 3962–3979. 10.1039/C7CS00169J. 28639669

[ref5] Polak D.; et al. Manipulating molecules with strong coupling: harvesting triplet excitons in organic exciton microcavities. Chem. Sci. 2020, 11, 343–354. 10.1039/C9SC04950A. 32190258 PMC7067247

[ref6] Eizner E.; Martínez-Martínez L. A.; Yuen-Zhou J.; Kéna-Cohen S. Inverting singlet and triplet excited states using strong light-matter coupling. Sci. Adv. 2019, 5, eaax4482 10.1126/sciadv.aax4482. 31840063 PMC6897552

[ref7] Climent C.; Galego J.; Garcia-Vidal F. J.; Feist J. Plasmonic Nanocavities Enable Self-Induced Electrostatic Catalysis. Angew. Chem., Int. Ed. 2019, 58, 8698–8702. 10.1002/anie.201901926. PMC6973273 30969014

[ref8] Thomas A.; George J.; Shalabney A.; Dryzhakov M.; Varma S. J.; Moran J.; Chervy T.; Zhong X.; Devaux E.; Genet C.; Hutchison J. A.; Ebbesen T. W. Ground-State Chemical Reactivity under Vibrational Coupling to the Vacuum Electromagnetic Field. Angew. Chem., Int. Ed. 2016, 55, 11462–11466. 10.1002/anie.201605504. PMC5113700 27529831

[ref9] Martínez-Martínez L. A.; Du M.; Ribeiro R. F.; Kéna-Cohen S.; Yuen-Zhou J. Polariton-Assisted Singlet Fission in Acene Aggregates. J. Phys. Chem. Lett. 2018, 9, 1951–1957. 10.1021/acs.jpclett.8b00008. 29551074

[ref10] Fregoni J.; Granucci G.; Persico M.; Corni S. Strong Coupling with Light Enhances the Photoisomerization Quantum Yield of Azobenzene. Chem. 2020, 6, 250–265. 10.1016/j.chempr.2019.11.001.

[ref11] Gu B.; Mukamel S. Manipulating nonadiabatic conical intersection dynamics by optical cavities. Chem. Sci. 2020, 11, 1290–1298. 10.1039/C9SC04992D. PMC8147895 34123253

[ref12] Antoniou P.; Suchanek F.; Varner J. F.; Foley J. J. Role of Cavity Losses on Nonadiabatic Couplings and Dynamics in Polaritonic Chemistry. J. Phys. Chem. Lett. 2020, 11, 9063–9069. 10.1021/acs.jpclett.0c02406. 33045837

[ref13] Mauro L.; Caicedo K.; Jonusauskas G.; Avriller R. Charge-transfer chemical reactions in nanofluidic Fabry-Pérot cavities. Phys. Rev. B: Condens. Matter Mater. Phys. 2021, 103, 165412 10.1103/PhysRevB.103.165412.

[ref14] Coccia E.; Corni S. Role of coherence in the plasmonic control of molecular absorption. J. Chem. Phys. 2019, 151, 044703 10.1063/1.5109378. 31370514

[ref15] Galego J.; Garcia-Vidal F. J.; Feist J. Cavity-Induced Modifications of Molecular Structure in the Strong-Coupling Regime. Phys. Rev. X 2015, 5, 041022 10.1103/PhysRevX.5.041022.

[ref16] Kowalewski M.; Bennett K.; Mukamel S. Non-adiabatic dynamics of molecules in optical cavities. J. Chem. Phys. 2016, 144, 054309 10.1063/1.4941053. 26851923

[ref17] Neuman T.; Esteban R.; Casanova D.; García-Vidal F. J.; Aizpurua J. Coupling of Molecular Emitters and Plasmonic Cavities beyond the Point-Dipole Approximation. Nano Lett. 2018, 18, 2358–2364. 10.1021/acs.nanolett.7b05297. 29522686

[ref18] Doppagne B.; Neuman T.; Soria-Martinez R.; López L. E. P.; Bulou H.; Romeo M.; Berciaud S.; Scheurer F.; Aizpurua J.; Schull G. Single-molecule tautomerization tracking through space- and time-resolved fluorescence spectroscopy. Nat. Nanotechnol. 2020, 15, 207–211. 10.1038/s41565-019-0620-x. 31959932

[ref19] Kosik M.; Burlayenko O.; Rockstuhl C.; Fernandez-Corbaton I.; Słowik K. Interaction of atomic systems with quantum vacuum beyond electric dipole approximation. Sci. Rep. 2020, 10, 5879 10.1038/s41598-020-62629-0. 32246018 PMC7125098

[ref20] Ojambati O. S.; Chikkaraddy R.; Deacon W. D.; Horton M.; Kos D.; Turek V. A.; Keyser U. F.; Baumberg J. J. Quantum electrodynamics at room temperature coupling a single vibrating molecule with a plasmonic nanocavity. Nat. Commun. 2019, 10, 1049 10.1038/s41467-019-08611-5. 30837456 PMC6400948

[ref21] Chen X.; Liu P.; Hu Z.; Jensen L. High-resolution tip-enhanced Raman scattering probes sub-molecular density changes. Nat. Commun. 2019, 10, 2567 10.1038/s41467-019-10618-x. 31189893 PMC6561954

[ref22] Haugland T. S.; Ronca E.; Kjønstad E. F.; Rubio A.; Koch H. Coupled Cluster Theory for Molecular Polaritons: Changing Ground and Excited States. Phys. Rev. X 2020, 10, 041043 10.1103/PhysRevX.10.041043.

[ref23] Haugland T. S.; Schäfer C.; Ronca E.; Rubio A.; Koch H. Intermolecular interactions in optical cavities: An ab initio QED study. J. Chem. Phys. 2021, 154, 094113 10.1063/5.0039256. 33685159

[ref24] Buhmann S. Y.; Butcher D. T.; Scheel S. Macroscopic quantum electrodynamics in nonlocal and nonreciprocal media. New J. Phys. 2012, 14, 083034 10.1088/1367-2630/14/8/083034.

[ref25] Feist J.; Fernández-Domínguez A. I.; García-Vidal F. J. Macroscopic QED for quantum nanophotonics: emitter-centered modes as a minimal basis for multiemitter problems. Nanophotonics 2020, 10, 477–489. 10.1515/nanoph-2020-0451.

[ref26] Helgaker T.; Jørgensen P.; Olsen J. Molecular Electronic-Structure Theory; John Wiley & Sons, Ltd: Chichester, U.K., 2000.

[ref27] Jaynes E. T.; Cummings F. W. Comparison of quantum and semiclassical radiation theories with application to the beam maser. Proc. IEEE 1963, 51, 89 10.1109/PROC.1963.1664.

[ref28] Sinha-Roy R.; García-González P.; Weissker H.-C.; Rabilloud F.; Fernández-Domínguez A. I. Classical and ab Initio Plasmonics Meet at Sub-nanometric Noble Metal Rods. ACS Photonics 2017, 4, 1484–1493. 10.1021/acsphotonics.7b00254.

[ref29] Urbieta M.; Barbry M.; Zhang Y.; Koval P.; Sánchez-Portal D.; Zabala N.; Aizpurua J. Atomic-Scale Lightning Rod Effect in Plasmonic Picocavities: A Classical View to a Quantum Effect. ACS Nano 2018, 12, 585–595. 10.1021/acsnano.7b07401. 29298379

[ref30] Yang B.; Chen G.; Ghafoor A.; Zhang Y.; Zhang Y.; Zhang Y.; Luo Y.; Yang J.; Sandoghdar V.; Aizpurua J.; Dong Z.; Hou J. G. Sub-nanometre resolution in single-molecule photoluminescence imaging. Nat. Photonics 2020, 14, 693–699. 10.1038/s41566-020-0677-y.

[ref31] Judge A. C.; Steel M. J.; Sipe J. E.; de Sterke C. M. Canonical quantization of macroscopic electrodynamics in a linear, inhomogeneous magnetoelectric medium. Phys. Rev. A: At., Mol., Opt. Phys. 2013, 87, 033824 10.1103/PhysRevA.87.033824.

[ref32] Mennucci B.; Corni S. Multiscale modelling of photoinduced processes in composite systems. Nat. Rev. Chem. 2019, 3, 315–330. 10.1038/s41570-019-0092-4.

[ref33] Kelly K. L.; Coronado E.; Zhao L. L.; Schatz G. C. The Optical Properties of Metal Nanoparticles: The Influence of Size, Shape, and Dielectric Environment. J. Phys. Chem. B 2003, 107, 668–677. 10.1021/jp026731y.

[ref34] Cherqui C.; Thakkar N.; Li G.; Camden J. P.; Masiello D. J. Characterizing Localized Surface Plasmons Using Electron Energy-Loss Spectroscopy. Annu. Rev. Phys. Chem. 2016, 67, 331–357. 10.1146/annurev-physchem-040214-121612. 27215817

[ref35] Trügler A.; Hohenester U. Strong coupling between a metallic nanoparticle and a single molecule. Phys. Rev. B: Condens. Matter Mater. Phys. 2008, 77, 115403 10.1103/PhysRevB.77.115403.

[ref36] Cancès E.; Mennucci B.; Tomasi J. A new integral equation formalism for the polarizable continuum model: Theoretical background and applications to isotropic and anisotropic dielectrics. J. Chem. Phys. 1997, 107, 3032–3041. 10.1063/1.474659.

[ref37] Corni S.; Pipolo S.; Cammi R. Equation of Motion for the Solvent Polarization Apparent Charges in the Polarizable Continuum Model: Application to Real-Time TDDFT. J. Phys. Chem. A 2015, 119, 5405–5416. 10.1021/jp5106828. 25485456

[ref38] Boyd R. W. Nonlinear Optics, 3rd ed.; Academic Press, Inc., 2008; pp 135–171.

[ref39] Guido C. A.; Rosa M.; Cammi R.; Corni S. An open quantum system theory for polarizable continuum models. J. Chem. Phys. 2020, 152, 174114 10.1063/5.0003523. 32384839

[ref40] Kristensen K.; Kauczor J.; Thorvaldsen A. J.; Jorgensen P.; Kjaergaard T.; Rizzo A. Damped response theory description of two-photon absorption. J. Chem. Phys. 2011, 134, 214104 10.1063/1.3595280. 21663341

[ref41] Delga A.; Feist J.; Bravo-Abad J.; Garcia-Vidal F. J. Quantum Emitters Near a Metal Nanoparticle: Strong Coupling and Quenching. Phys. Rev. Lett. 2014, 112, 253601 10.1103/PhysRevLett.112.253601. 25014814

[ref42] Flick J.; Ruggenthaler M.; Appel H.; Rubio A. Atoms and molecules in cavities, from weak to strong coupling in quantum-electrodynamics (QED) chemistry. Proc. Natl. Acad. Sci. U. S. A. 2017, 114, 3026–3034. 10.1073/pnas.1615509114. 28275094 PMC5373338

[ref43] Schäfer C.; Ruggenthaler M.; Rubio A. Ab initio nonrelativistic quantum electrodynamics: Bridging quantum chemistry and quantum optics from weak to strong coupling. Phys. Rev. A: At., Mol., Opt. Phys. 2018, 98, 043801 10.1103/PhysRevA.98.043801.

[ref44] Novotny L.; Hecht B. Principles of Nano-Optics; Cambridge University Press, 2006.

[ref45] Corni S.; Tomasi J. Enhanced response properties of a chromophore physisorbed on a metal particle. J. Chem. Phys. 2001, 114, 3739–3751. 10.1063/1.1342241.

[ref46] Tomasi J.; Mennucci B.; Cammi R. Quantum Mechanical Continuum Solvation Models. Chem. Rev. 2005, 105, 2999–3094. 10.1021/cr9904009. 16092826

[ref47] Jensen L. L.; Jensen L. Atomistic Electrodynamics Model for Optical Properties of Silver Nanoclusters. J. Phys. Chem. C 2009, 113, 15182–15190. 10.1021/jp904956f.

[ref48] Chen X.; Liu P.; Jensen L. Atomistic electrodynamics simulations of plasmonic nanoparticles. J. Phys. D: Appl. Phys. 2019, 52, 363002 10.1088/1361-6463/ab249d.

[ref49] Giovannini T.; Rosa M.; Corni S.; Cappelli C. A classical picture of subnanometer junctions: an atomistic Drude approach to nanoplasmonics. Nanoscale 2019, 11, 6004–6015. 10.1039/C8NR09134J. 30869089

[ref50] Baranov D. G.; Munkhbat B.; Zhukova E.; Bisht A.; Canales A.; Rousseaux B.; Johansson G.; Antosiewicz T. J.; Shegai T. Ultrastrong coupling between nanoparticle plasmons and cavity photons at ambient conditions. Nat. Commun. 2020, 11, 2715 10.1038/s41467-020-16524-x. 32483151 PMC7264206

