# Supplementary material for: Strong Coupling between Localized Surface Plasmons and Molecules by Coupled Cluster Theory
Source: Nano Lett. 2021 Jul 20;21(15):6664–70. doi: 10.1021/acs.nanolett.1c02162 (PMC8361433; doi:10.1021/acs.nanolett.1c02162)
Supplement: Supplementary file 1 — nl1c02162_si_001.pdf [file nl1c02162_si_001.pdf]

Supporting Information: Strong coupling between localized surface plasmons and molecules by coupled cluster theory

Jacopo Fregoni*,[†,‡] Tor S. Haugland*,[¶] Silvio Pipolo,[§] Tommaso Giovannini,[||] Henrik Koch,[*,||,¶] and Stefano Corni*,[†,‡]

†*Dipartimento di Scienze Chimiche, University of Padova, I-35131 Padova, Italy*

‡*Istitute of Nanosciences, Consiglio Nazionale delle Ricerche CNR-Nano, I-41125 Modena, Italy*

¶*Department of Chemistry, Norwegian University of Science and Technology, 7491 Trondheim, Norway*

§*UCCS Unité de Catalyse et Chimie du Solide, Université de Lille, Université d'Artois UMR 8181, F-59000, Lille, France*

||*Scuola Normale Superiore, I-56126, Pisa, Italy*

E-mail: henrik.koch@sns.it; stefano.corni@unipd.it

[*] These authors contributed equally to the realization of the present work

Q-PCM-NP

Response of the plasmonic nanostructures to an electromagnetic field: the PCM-NP equations in the diagonal formulation

The first step is to choose a convenient description of the classical dielectric response of the nanoparticle. Our approach builds on the PCM-NP formalism.[1,2] In particular, we make use of PCM relations from the integral equation formalism.[3]

To classically compute the nanoparticle surface properties means to classically solve Maxwell's equations for a continuous, frequency-dependent dielectric (nanostructure) under an external perturbation. Put differently, simulating the plasmons of a nanoparticle calls for the computation of the nanoparticle's linear response to the external perturbation. In the quasi-static limit, the nanoparticle experiences an electrostatic potential $\boldsymbol{V}(\omega)$ acting on the nanoparticle surface, where ω is the frequency associated to the external potential (*e.g.* produced by a molecular transition). Such potential induces polarisation charges. A commonly adopted solution to the electrostatic problem is to resort to discretisation techniques (Figures S1a and S1b) such as the boundary element method (BEM).[1,4–7]

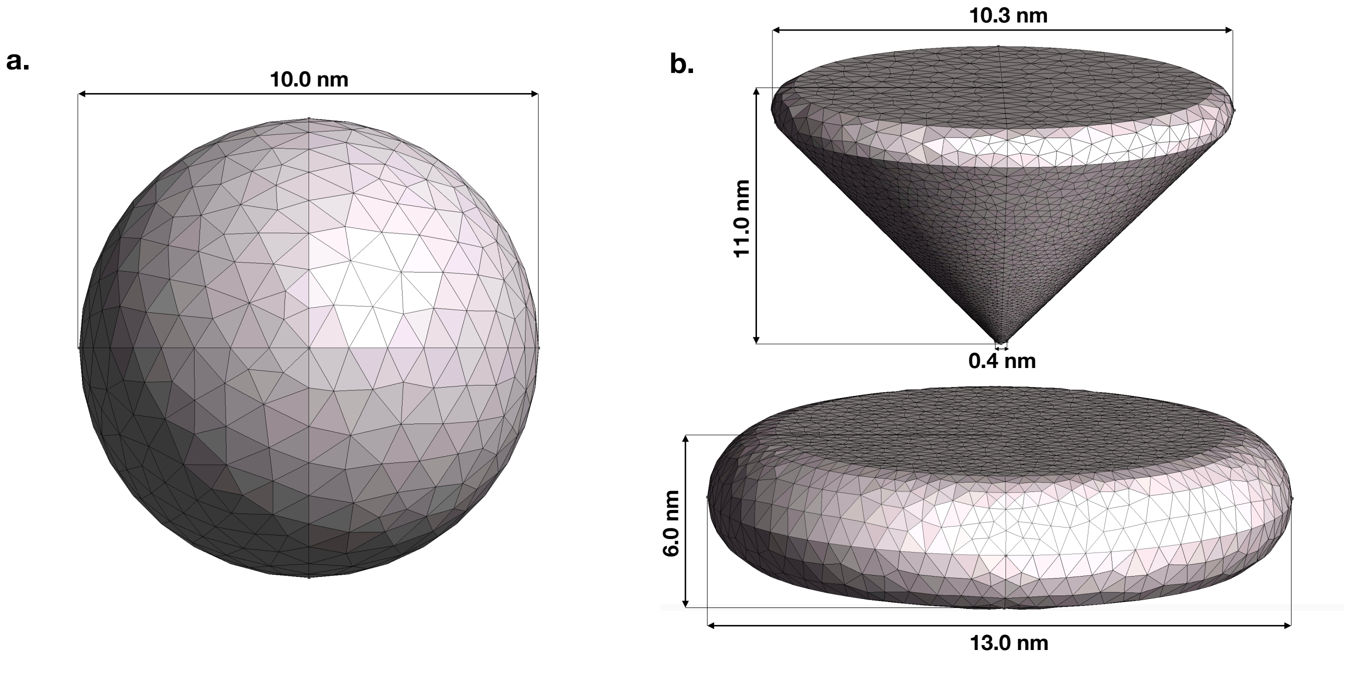

Figure S1: **Nanoparticles surfaces discretised with BEM| a)** Surface discretization of a 10 nm spherical nanoparticle, which will be used in the tests of the presented method. **b)** Discretisation of a nanotip setup. The dimensions are reproduced after ref.[8]

Within such formulation, a set of apparent surface charges (ASC) sitting on the representative points of the nanoparticle discretised surface is used to represent the electrostatic potential that solves the Poisson

equation. We can consider the ASC as the representation of the linear dielectric response of the nanoparticle to the external perturbation at different ω. The IEF-BEM equation defining the ASC ($\boldsymbol{q}$) is

$$\boldsymbol{q}(\omega) = \boldsymbol{Q}^{IEF}(\omega)\boldsymbol{V}(\omega) \tag{S1}$$

where $\boldsymbol{Q}^{IEF}(\omega)$ is the frequency-dependent response matrix to the external perturbation $\boldsymbol{V}(\omega)$. It describes the redistribution of the surface charges in the NP when the system is subjected to an external perturbation which, here, will be the electric field associated to the molecular transition density. $\boldsymbol{Q}^{IEF}(\omega)$ is computed following the standard IEF formalism as:[9]

$$\boldsymbol{Q}^{IEF}(\omega) = -\boldsymbol{S}^{-1}\left(2\pi\frac{\varepsilon(\omega)+1}{\varepsilon(\omega)-1}\boldsymbol{I} + \boldsymbol{DA}\right)^{-1}(2\pi\boldsymbol{I} + \boldsymbol{DA}). \tag{S2}$$

Here, $\varepsilon(\omega)$ is the dielectric function of the medium, $\boldsymbol{A}$ is a diagonal matrix containing the area of each discrete surface element (tessera), while $\boldsymbol{S}$ and $\boldsymbol{D}$ are the matrix representations of Calderon's projectors, respectively storing information on the electrostatic potentials and the electric field between charges sitting on different tesserae:

$$S_{ij} = \frac{1}{|\boldsymbol{s_i} - \boldsymbol{s_j}|} \qquad D_{ij} = \frac{(\boldsymbol{s_i} - \boldsymbol{s_j}) \cdot \boldsymbol{n_j}}{|\boldsymbol{s_i} - \boldsymbol{s_j}|^3}, \tag{S3}$$

for the out of diagonal elements (for diagonal elements see, e.g., ref.[10]) where $\boldsymbol{s_i}$ and $\boldsymbol{s_j}$ collect the coordinates of the $i-$th and $j-$th tessera and $\boldsymbol{n_j}$ is the unit vector directed along the normal of the $j-$th tessera. Examining the response function in eq. S2, the frequency dependence is embodied in the choice of the dielectric function $\varepsilon(\omega)$. Following the procedure outlined in previous works,[2] it is possible to reformulate the matrix in a diagonal form:

$$\boldsymbol{Q}^{IEF}(\omega) = -\boldsymbol{S}^{-\frac{1}{2}}\boldsymbol{T}\left(2\pi\frac{\varepsilon(\omega)+1}{\varepsilon(\omega)-1}\boldsymbol{I} + \boldsymbol{\Lambda}\right)^{-1}(2\pi\boldsymbol{I} + \boldsymbol{\Lambda})\,\boldsymbol{T}^{\dagger}\boldsymbol{S}^{-\frac{1}{2}}, \tag{S4}$$

Here, the diagonal matrix $\boldsymbol{\Lambda}$ and the matrix $\boldsymbol{T}$ collect the eigenvalues and eigenvectors of $\boldsymbol{S}^{-\frac{1}{2}}\boldsymbol{DAS}^{\frac{1}{2}}$, which is (formally) symmetric. A compact form to indicate the diagonalized version of $\boldsymbol{Q}^{IEF}(\omega)$ is obtained when collecting all the central terms of eq. S4 into a diagonal matrix $\boldsymbol{K}(\omega)$:

$$\boldsymbol{Q}^{IEF}(\omega) = -\boldsymbol{S}^{-\frac{1}{2}}\boldsymbol{TK}(\omega)\boldsymbol{T}^{\dagger}\boldsymbol{S}^{-\frac{1}{2}}, \tag{S5}$$

$$K_p(\omega) = \frac{2\pi + \Lambda_p}{2\pi\frac{\varepsilon(\omega)+1}{\varepsilon(\omega)-1} + \Lambda_p}. \tag{S6}$$

K_p and Λ_p are the respective diagonal elements of the $\boldsymbol{K}(\omega)$ and $\boldsymbol{\Lambda}$ matrices. By substituting in the expression above the Drude-Lorentz dielectric function $\varepsilon(\omega)$:

$$\varepsilon(\omega) = 1 + \frac{\Omega_P^2}{\omega_0^2 - \omega^2 - i\gamma\omega}, \tag{S7}$$

we make the frequency dependence of K_p explicit:

$$K_p(\omega) = \frac{\left(1+\frac{\Lambda_p}{2}\right)\frac{\Omega_P^2}{2}}{\omega_0^2 - \omega^2 - i\gamma\omega + \left(1+\frac{\Lambda_p}{2\pi}\right)\frac{\Omega_P^2}{2}}. \tag{S8}$$

We define the plasmon frequency as $\omega_p^2 = \omega_0^2 + \left(1+\frac{\Lambda_p}{2\pi}\right)\frac{\Omega_P^2}{2}$ (where we have neglected a second-order term in γ) and obtain

$$K_p(\omega) = \frac{\omega_p^2 - \omega_0^2}{\omega_p}\frac{\omega_p}{\left(\omega_p - \omega - i\frac{\gamma}{2}\right)\left(\omega_p + \omega + i\frac{\gamma}{2}\right)} \tag{S9}$$

$$= \frac{\omega_p^2 - \omega_0^2}{2\omega_p}\left(\frac{1}{\left(\omega_p + \omega + i\frac{\gamma}{2}\right)} + \frac{1}{\left(\omega_p - \omega - i\frac{\gamma}{2}\right)}\right). \tag{S10}$$

By making use of this form for $K_p(\omega)$ and of eq. S5, we retrieve the full form of the response function $\boldsymbol{Q}^{IEF}(\omega)$ as:

$$\boldsymbol{Q}_{kj}^{IEF}(\omega) = -\sum_p \left(\boldsymbol{S}^{-\frac{1}{2}}\boldsymbol{T}\right)_{k,p}\sqrt{\frac{\omega_p^2 - \omega_0^2}{2\omega_p}}\left(\frac{1}{\omega_p + \omega + i\frac{\gamma}{2}} + \frac{1}{\omega_p - \omega - i\frac{\gamma}{2}}\right)\sqrt{\frac{\omega_p^2 - \omega_0^2}{2\omega_p}}\left(\boldsymbol{T}^\dagger \boldsymbol{S}^{-\frac{1}{2}}\right)_{p,j}. \tag{S11}$$

Correspondence between macroscopic QED charges and Q-PCM-NP: the Drude dielectric limit

In this section, we compare the Drude limit ($\omega_0 = 0$) of the Q-PCM-NP formulation to the macroscopic QED charges. To do so, we report eq. 8 from the main text, representing the set of response charges associated to a plasmonic mode p:

$$\langle 0|\hat{q}_k|p\rangle = \left(\boldsymbol{S}^{-\frac{1}{2}}\boldsymbol{T}\right)_{k,p}\sqrt{\frac{\omega_p^2 - \omega_0^2}{2\omega_p}} = q_{p,k}. \tag{S12}$$

The individual sets of charges describing the quantised plasmons with the Q-PCM-NP method are analogous to the ones obatined in ref.,[11] based on macroscopic QED for a Drude dielectric. There, the charges are defined via the right eigenvectors $\sigma_p(\mathbf{s})$ of an integral operator kernel $\mathcal{F}(s, s')$ that, once integral equations are discretized, is equivalent to $\mathbf{D}^\dagger$ in our notation. More specifically

$$\int_{\partial V_{pl}} \mathcal{F}(\mathbf{s}, \mathbf{s}')\sigma_p(\mathbf{s}')\, d^2\mathbf{s}' = \lambda_p \sigma_p(\mathbf{s}) \tag{S13}$$

with (in our notation and using atomic units):

$$\int_{\partial V_{pl}}\int_{\partial V_{pl}} \frac{\sigma_p^*(\mathbf{s}')\sigma_p(\mathbf{s})}{|\mathbf{s}-\mathbf{s}'|} d^2\mathbf{s}\, d^2\mathbf{s}' = \frac{1}{2}\sqrt{\frac{\Omega_P^2}{2}(1+\frac{\lambda_p}{2\pi})} \tag{S14}$$

where ∂V_{pl} is the surface of the nanoparticle. In the discretized version and the present paper notation, such equations read:

$$\mathbf{A}\mathbf{D}^\dagger \mathbf{q}_p = \lambda_p \mathbf{q}_p \tag{S15}$$

$$\mathbf{q}_p^\dagger \mathbf{S}\mathbf{q}_p = \frac{1}{2}\sqrt{\frac{\Omega_P^2}{2}(1+\frac{\lambda_p}{2\pi})} \tag{S16}$$

By multiplying eq.(S15) on the left by $\mathbf{S}^{1/2}$, and using $\mathbf{S}^{1/2}\mathbf{A}\mathbf{D}^\dagger S^{-1/2} = \mathbf{S}^{-1/2}\mathbf{D}\mathbf{A}\mathbf{S}^{1/2}$ we get:

$$\mathbf{S}^{-1/2}\mathbf{D}\mathbf{A}\mathbf{S}^{1/2}\mathbf{S}^{1/2}\mathbf{q}_p = \lambda_p \mathbf{S}^{1/2}\mathbf{q}_p \tag{S17}$$

Λ_p and the p column of the matrix $\mathbf{T}$ in the main text are by construction the eigenvalue and the corresponding eigenvector of the matrix $\mathbf{S}^{-1/2}\mathbf{D}\mathbf{A}\mathbf{S}^{1/2}$.[2] Therefore, $\lambda_p = \Lambda_p$, $\mathbf{q}_p^\dagger \mathbf{S}\mathbf{q}_p = \frac{1}{2}\omega_p$ for Drude model ($\omega_0 = 0$) and $\alpha_p \mathbf{S}^{1/2}\mathbf{q}_p = \mathbf{T}_p$, where α_p is a normalization factor to be determined, that can be taken real. To obtain the latter, we note that:

$$1 = \mathbf{T}_p^\dagger \mathbf{T}_p = \alpha_p^2 \mathbf{q}_p^\dagger \mathbf{S}\mathbf{q}_p = \alpha_p^2 \frac{1}{2}\omega_p \tag{S18}$$

Therefore, $\alpha_p = \sqrt{2/\omega_p}$ and $\sqrt{2/\omega_p}\mathbf{S}^{1/2}\mathbf{q}_p = \mathbf{T}_p$, that is:

$$\mathbf{q}_p = \sqrt{\frac{\omega_p}{2}}\mathbf{S}^{-1/2}\mathbf{T}_p \tag{S19}$$

that is the expression given in eq. S20 of the main text, once it is reduced to the Drude model ($\omega_0 = 0$). Therefore, our result does match the $\mathbf{q}_p$ from ref.[11] in the case of a Drude metal.

Simplified coupling models

In the main text we described how fully correlated plasmon-molecules wave functions can be obtained with QED-CCSD-1. In this section we shall derive approximate expressions of the plasmon-molecule coupling strength based on the properties of the unperturbed gas-phase molecule, and then by further reducing it to a point dipole. The former is an ubiquitous assumption in the theoretical descriptions of molecule-plasmonic nanostructrure strong coupling, whose consequences we are here in the position to check for the first time. The latter is a common assumption, whose general validity has been already questioned in the field of strong coupling,[11] and before in the field of surface enhanced spectroscopies.[1,12]

In eq. 9 of the main text, we show the plasmon-molecule interaction in the quasi-static limit. Based on such

equation, we define - for each plasmonic mode p - the plasmon-molecule coupling operator $\hat{g}_p$ as

$$\hat{g}_p = \sum_j q_{p,j} \hat{V}_j, \tag{S20}$$

hence reducing the interaction Hamiltonian to:

$$\hat{H}_{int} = \sum_p \hat{g}_p \left(\hat{b}_p^\dagger + \hat{b}_p \right). \tag{S21}$$

The simplest approximation to the plasmon-molecule coupling strength g_p for a specific plasmon mode p is defined by the matrix elements of $\hat{H}_{int}$ on the minimal basis of non-interacting states required to describe the polariton formation, i.e., $|S_0\rangle \otimes |1\rangle = |S_0, 1\rangle$ and $|S_1\rangle \otimes |0\rangle = |S_1, 0\rangle$. S_0 and S_1 are the ground and the first excited state of the molecule, respectively; $|0\rangle$ and $|1\rangle$ are the plasmonic states characterized by the occupation number of the plasmonic mode we are focusing on (either 0 or 1 in this example). Using eq. S20, one gets:

$$g_p^{bem,full} = \left\langle S_0, 1 \left| \hat{H}_{int} \right| S_1, 0 \right\rangle = \left\langle S_0, 1 \left| \hat{g}_p \hat{b}^\dagger \right| S_1, 0 \right\rangle = \sum_j q_{p,j} V_j^{(S_0,S_1)}, \tag{S22}$$

where $V_j^{(S_0,S_1)}$ is the potential originated by the $S_0 \longrightarrow S_1$ transition on the j-th tessera. The superscript $bem, full$ reminds us that this is an expression obtained by the *BEM* formulation with *full* consideration of the spatial extent of the molecule, i.e., no approximation of the molecule as a point dipole. To find an expression for $V_j^{(S_0,S_1)}$, it is convenient to describe the local behaviour of the molecule in terms of one-particle operators in second quantization. The potential acting on the j-th tessera is then the potential associated to the one-particle electron density operator $\hat{\rho}(\boldsymbol{r})$, written in terms of the molecular orbitals $\phi_r(\boldsymbol{r})$, $\phi_s(\boldsymbol{r})$:

$$\hat{V}_j = \int_{Vol} d^3\boldsymbol{r} \frac{\hat{\rho}(\boldsymbol{r})}{|\boldsymbol{s_j} - \boldsymbol{r}|} = \sum_{rs} \int_{Vol} d^3\boldsymbol{r} \frac{\rho_{rs}(\boldsymbol{r}) \hat{a}_r^\dagger \hat{a}_s}{|\boldsymbol{s_j} - \boldsymbol{r}|} = \sum_{rs} V_j^{rs} \hat{a}_r^\dagger \hat{a}_s. \tag{S23}$$

Consequently, the molecule-nanoparticle coupling operator $\hat{g}_p$ defined in eq.(17 of the main text) can be obtained as:

$$\hat{g}_p = \sum_{rs} q_{p,j} V_j^{rs} \hat{a}_r^\dagger \hat{a}_s = \sum_{rs} g_p^{rs} \hat{a}_r^\dagger \hat{a}_s. \tag{S24}$$

with

$$g_p^{rs} = \sum_j q_{p,j} V_j^{rs} \tag{S25}$$

The coupling element between the $S_0 \longrightarrow S_1$ transition is then evaluated as:

$$g_p^{bem,full} = \left\langle S_0, 1 \left| \sum_{rs} g_p^{rs} \hat{a}_r^\dagger \hat{a}_s \hat{b}^\dagger \right| S_1, 0 \right\rangle = \sum_{rs} \rho_{rs}^{(S_0,S_1)} g_p^{rs}, \tag{S26}$$

where we made use of the transition density matrix $\rho_{rs}^{(S_0,S_1)}$ to retrieve a similar formalism to the one proposed

in refs.[8,11] The expression of $g_p^{bem,full}$ can be simplified by considering the molecule as a point-dipole and retrieve therefore a Jaynes-Cummings-like[13] description of the interaction between the nanoparticle and the molecule.[14–16] For a point-dipole molecule:

$$g_p^{bem,dip} = \left\langle S_0, 1 \left| \sum_j q_{p,j} \frac{(\boldsymbol{s_j} - \boldsymbol{r_d}) \cdot \boldsymbol{\mu}^{S_0,S_1}}{|\boldsymbol{s_j} - \boldsymbol{r_d}|^3} \hat{b}_p^\dagger \right| S_1, 0 \right\rangle = E_{q_p} \boldsymbol{\lambda_{q_p}} \cdot \boldsymbol{\mu}^{(S_0,S_1)} \tag{S27}$$

Here, $\boldsymbol{\mu}^{(S_0,S_1)}$ is the molecular transition dipole and $E_{q_p}\boldsymbol{\lambda_{q_p}}$ (where $\boldsymbol{\lambda_{q_p}}$ is the polarisation unit vector), is the electric field associated to the apparent surface charges of the plasmonic mode under investigation $\boldsymbol{q_p}$:

$$E_{q_p} \boldsymbol{\lambda_{q_p}} = \sum_j q_{p,j} \frac{(\boldsymbol{s_j} - \boldsymbol{r_d})}{|\boldsymbol{s_j} - \boldsymbol{r_d}|^3} \tag{S28}$$

where $\boldsymbol{s_j} - \boldsymbol{r_d}$ is the distance between the tessera representative point $\boldsymbol{s_j}$ and the position of the point dipole $\boldsymbol{r_d}$. This expression directly connects to the typical $\hat{H}_{int}$ form used in approximated QED treatments where only the field-dipole term is retained, i.e.:

$$\hat{H}_{int}^{dip} = \sum_p E_{1ph,p} \boldsymbol{\lambda_p} \cdot \hat{\boldsymbol{\mu}} \left(\hat{b}_p^\dagger + \hat{b}_p \right). \tag{S29}$$

For simple geometries, such as a nanosphere, the $E_{1ph,p}$ coefficient (E_{q_p} when calculated from BEM) can be obtained analytically,[17] leading to another estimate of the coupling strength:

$$g_p^{an,dip} = E_{1ph,p} \left\langle S_0, 1 \left| \hat{g}_p^{dip} \hat{b}_p^\dagger \right| S_1, 0 \right\rangle = E_{1ph,p} \boldsymbol{\lambda_p} \cdot \boldsymbol{\mu_{S_0,S_1}} \tag{S30}$$

The three simplified expressions introduced here, $g_p^{an,dip}$, $g_p^{bem,dip}$ and $g_p^{bem,full}$ are represented and compared in Figures S2 and S3. In the Results section of the main text we instead focus on applying this formalism to the case of realistic molecules, where we also provide polaritonic absorption spectra calculated with the full QED-CCSD-1 model and by considering polaritons obtained by a simple diagonalization of the system Hamiltonian in the $|S_0, 1\rangle$ and $|S_1, 0\rangle$ subspace, with $g_p^{bem,full}$ as the non-diagonal matrix element.

QED coupled cluster algorithm

The results of this paper are calculated by coupling multiple advanced algorithms. Here we outline how the calculations have been performed and self-consistency reached. From the geometry of the nanoparticle, the plasmon modes and corresponding charges and excitation energies are determined from the Drude-Lorentz parameters using the method outlined in the main text. This corresponds to determining the plasmonic basis. We write the plasmonic wave function as a linear combination of the modes p:

$$|P\rangle = \sum_{\vec{n}} \prod_p (b_p^\dagger)^{n_p} |0\rangle c_{\vec{n}} \tag{S31}$$

where we recognize the meaning of $|0\rangle$ as the plasmon vacuum and $\vec{n} = (n_1, n_2, \dots)$ is a vector collecting the plasmon occupation numbers in each mode

As presented in the main text, the electron-plasmon Hamiltonian reads:

$$\hat{H} = \hat{H}_e + \sum_p \omega_p \hat{b}_p^\dagger \hat{b}_p + \sum_p \sum_j q_{p,j} \hat{V}_j \left(\hat{b}_p^\dagger + \hat{b}_p \right). \tag{S32}$$

The electrostatic potential operator $\hat{V}_j$ is defined in eq. S23. We now have a form of the Hamiltonian that resembles the Pauli-Fierz Hamiltonian presented by Haugland et al.[18] As detailed in the same reference, the non-correlated wave function is written as:

$$|R\rangle = |HF\rangle \otimes |P\rangle. \tag{S33}$$

The plasmon charges $q_{p,j}$ and energy ω_P are input to QED-HF and the energy to the combined electron-plasmon system is minimized. The problem is solved in a self-consistent field until the coefficients of the electronic orbitals and plasmon number states reach self-consistency. The result of this initial calculation yields the QED-HF reference state, and we report the main steps presented in ref.[18] for our current Hamiltonian. The energy minimization with respect to the plasmonic coefficients is achieved by diagonalizing the Hamiltonian in eq. S32. By applying the unitary coherent-state transformation:

$$U(\mathbf{z}) = \prod_p \exp(z_p(\hat{b}_p^\dagger - \hat{b}_p)), \tag{S34}$$

we obtain

$$\langle HF|U^\dagger(\mathbf{z})\hat{H}U(\mathbf{z})|HF\rangle = E_{HF} + \sum_p \left(\omega_p(\hat{b}_p^\dagger + z_p)(\hat{b}_p + z_p) + \sum_j q_{pj}\langle \hat{V}_j \rangle (\hat{b}_p + \hat{b}_p^\dagger + 2z_p) \right). \tag{S35}$$

Choosing the coherent state parameters z_p as

$$z_p = -\sum_j \frac{q_{pj}\langle \hat{V}_j \rangle}{\omega_p}, \tag{S36}$$

we obtain in fact a diagonal Hamiltonian in the plasmonic number states (of the coherent state-transformed plasmon basis):

$$\langle HF|U^\dagger(\mathbf{z})\hat{H}U(\mathbf{z})|HF\rangle = E_{HF} + \sum_p \left(\omega_p \hat{b}_p^\dagger \hat{b}_p - \frac{1}{\omega_P} \sum_j q_{pj}\langle \hat{V}_j \rangle \sum_k q_{pk}\langle \hat{V}_k \rangle \right). \tag{S37}$$

The expression in this equation is then minimized with respect to the electronic degrees of freedom. In each iteration of the minimization procedure we update the $|HF\rangle$ orbitals and use them to compute $\langle \hat{V}_j \rangle$. The

Fock matrix for the orbital optimization now includes the plasmon-molecule interaction:

$$F_{rs} = F^{e}_{rs} - \sum_{p} \left(\frac{2}{\omega_p} \sum_{j} q_{pj} \langle \hat{V}_j \rangle \sum_{k} q_{pk} \hat{V}^{rs}_{k} \right), \tag{S38}$$

where r, s denote the orbitals and $\boldsymbol{F}^e$ is the standard Fock matrix for closed shell systems. The Fock matrix expression can be made physically more transparent by noticing that in view of eqs. 5 and 7 in the main text, it turns out that:

$$-\sum_{p} \frac{2}{\omega_p} q_{pj} q_{pk} = Q^{IEF}_{kj}(0) \tag{S39}$$

where $Q^{IEF}_{kj}(0)$ is the classical response matrix evaluated for a static perturbation ($\omega = 0$). This leads to:

$$F_{rs} = F^{e}_{rs} + \sum_{jk} Q^{IEF}_{kj}(0) \langle \hat{V}_j \rangle \hat{V}^{rs}_{k}, \tag{S40}$$

Based on eq. 1 in the main text, $\sum_j Q^{IEF}_{kj}(0)\langle \hat{V}_j \rangle$ are the classical polarization charges q^{HF}_k induced on the plasmonic nanostructure due to the dielectric response to the molecule-generated electrostatic potential at the HF level. The Fock operator reduces then to:

$$F_{rs} = F^{e}_{rs} + \sum_{k} q^{HF}_{k} \hat{V}^{rs}_{k}. \tag{S41}$$

Like in classical implicit solvation theories,[19] the extra term in the Fock operator represents the interaction of the molecular electrons with the dielectric polarization induced in the metal nanostructure by the molecule itself. The iterative resolution of eq.S38 provides self-consistency of the molecular wave function and nanostructure polarization, for the plasmonic modes included in the calculation. Seen in the perspective of quantized plasmon states, in the calculation of the QED-HF reference state, the plasmonic states act as a basis for the polaritonic calculation, hence we optimize the coefficients of the plasmonic states, each of which is associated with a fixed set of charges $q_{p,j}$. This is reminiscent of how atomic orbitals are not optimized in an electronic structure calculation, it is their coefficients that are optimized.

After the reference QED-HF state is found, we obtain the coupled plasmon-molecule Hamiltonian:

$$\hat{H} = \hat{H}_e + \sum_{p} \left(\omega_p \hat{b}^{\dagger}_p \hat{b}_p + \sum_{j} q_{pj} \left(\hat{V}_j - \langle \hat{V}_j \rangle \right) \left(\hat{b}_p + \hat{b}^{\dagger}_p \right) + \frac{1}{\omega_p} \sum_{j} q_{pj} \langle \hat{V}_j \rangle \sum_{k} q_{pk} \left(\langle \hat{V}_k \rangle - 2\hat{V}_k \right) \right) \tag{S42}$$

where the expectation value of $\hat{V}_j$ is calculated with the HF wave function. At this stage, the QED-CCSD-1 ansatz is applied and the coupled cluster projection equations are solved to find the ground state. The excitation operators defined in the next section introduce correlation between the molecular and the plasmonic excitations, as well as readjust the plasmonic polarization to the CCSD molecular wave function.

QED coupled cluster (QED-CCSD) operators

Here, we provide explicit expressions for the coupled cluster operators. Starting from the molecule without plasmon, the wave function is chosen to be a single Slater determinant $|\mathrm{HF}\rangle$ (Hartree-Fock). Electron correlation is introduced through the exponential of the cluster operator acting on the $|\mathrm{HF}\rangle$ determinant,

$$|\mathrm{CC}\rangle = e^{\hat{T}}|\mathrm{HF}\rangle. \tag{S43}$$

The cluster operator $\hat{T}$ is expressed as

$$\hat{T} = \hat{T}_1 + \hat{T}_2 + \cdots + \hat{T}_{N_e} \tag{S44}$$

where T_1 are linear combinations of single excitations, T_2 are linear combinations of double excitations and so on. More specifically, T_1 and T_2 is given by

$$\hat{T}_1 = \sum_{ai} t_{ai} \sum_{\sigma} \hat{a}^\dagger_{a\sigma}\hat{a}_{i\sigma} \tag{S45}$$

$$\hat{T}_2 = \frac{1}{2}\sum_{aibj} t_{aibj} \sum_{\sigma\tau} \hat{a}^\dagger_{a\sigma}\hat{a}_{i\sigma}\hat{a}^\dagger_{b\tau}\hat{a}_{j\tau} \tag{S46}$$

where a, b and i, j are indices refer to virtual and occupied orbitals respectively. The operators $\hat{a}^\dagger$, a refer to the standard second quantization notation adopted in electronic structure theory. The coupled cluster method is equivalent to the exact diagonalization in the limit where all excitations are included in $\hat{T}$. Instead of formulating exact diagonalization in a more complicated manner, one truncates the cluster operator to $\hat{T}_1 + \hat{T}_2$ with the aim of reducing the computational complexity. This truncated approach is named CCSD, and for more details we refer to ref.[20]

In QED-CCSD-1, the cluster operator is limited to one plasmon with one plasmon mode,

$$\hat{T} = \hat{T}_1 + \hat{T}_2 + \hat{S}^1_1 + \hat{S}^1_2 + \hat{\Gamma}^1. \tag{S47}$$

Each term is a linear combination of excitation operators,

$$\hat{S}^1_1 = \sum_{ai} s_{ai} \sum_{\sigma} \hat{a}^\dagger_{a\sigma}\hat{a}_{i\sigma}\hat{b}^\dagger_p, \tag{S48}$$

$$\hat{S}^1_2 = \frac{1}{2}\sum_{aibj} s_{aibj} \sum_{\sigma\tau} \hat{a}^\dagger_{a\sigma}\hat{a}_{i\sigma}\hat{a}^\dagger_{b\tau}\hat{a}_{j\tau}\hat{b}^\dagger_p, \tag{S49}$$

$$\hat{\Gamma}^1 = \gamma_1\hat{b}^\dagger_p. \tag{S50}$$

Comparing CCSD and QED-CCSD-1 also includes the correlation contribution induced on the electronic states by the plasmonic mode without adding computational complexity: indeed, the QED-CCSD-1 has the same scaling with respect to the number of orbitals as in the CCSD case (N^6_{orb}), and is computationally

feasible for small to medium-sized molecules. In the limit where the interaction $\hat{g} \rightarrow 0$, these two methods are equivalent.

As mentioned in the SI section "Simplified coupling models", in this work we also provide approximated results akin to the JC model, although with a Quantum Chemistry derived coupling. The bilinear interaction is treated as a perturbation. Now one can use standard HF and CCSD instead of their QED equivalent and find the electronic structure independently from the plasmons, and then compute the bilinear interaction as a property, see Eq. (S26). In this case we form a basis from the ground state and a selected subset of excited electronic states together with the plasmon number states, and diagonalize the total Hamiltonian similarly to a Jaynes-Cummings model. The difference between the two methods being that in QED-CCSD, all singles and doubles states interact with the plasmons, but in Jaynes-Cummings CCSD, only a subset of states interact with the plasmon, usually through an approximate interaction.

Oscillator strengths

In this study, we use EOM-QED-CCSD-1 to compute the energies and oscillator strengths. The oscillator strengths f are computed as

$$f = \frac{2}{3}(E_e - E_g)|\langle e|\hat{d}_e + d_p(\hat{b}_p^\dagger + \hat{b}_p)|g\rangle|^2 \tag{S51}$$

where $\hat{d}_e$ is the electronic dipole moment operator and d_p is the plasmon dipole moment. Here g and e are the ground and excited states. The above expression is for symmetric theories, but since CCSD and QED-CCSD-1 are nonsymmetric, the expression is slightly modified,

$$f = \frac{2}{3}(E_e - E_g)\langle\Lambda|\hat{d}_e + d_p(\hat{b}_p^\dagger + \hat{b}_p)|R\rangle\langle L|\hat{d}_e + d_p(\hat{b}_p^\dagger + \hat{b}_p)|CC\rangle. \tag{S52}$$

Here $|R\rangle$ and $\langle L|$ are the right and left excited states of coupled cluster, $\langle\Lambda|$ is the left ground state and $|CC\rangle$ is the right coupled cluster ground state.

Supplementary Numerical Results

Numerical comparison of simplified plasmon-molecule coupling models

Here, we will compare the coupling between a spherical nanoparticle and a molecule obtained in three different ways of increasing accuracy, as sketched in Figure S2:

- Coupling between analytical spherical plasmonic modes and a point-dipole molecule (Figure S2a), $g_p^{an,dip}$. This approach was proposed by Delga *et al.*[17]
- Coupling between the plasmonic modes computed at Q-PCM-NP level and a point-dipole molecule (Figure S2b), $g_p^{bem,dip}$. This is done to directly compare with the analytical results.

- Coupling between the plasmonic modes computed at Q-PCM-NP level and a realistic molecule, computed at EOM-CCSD level (Figure S2c), $g_p^{bem,full}$. This is a central feature of this work.

As a test molecule, we take hydrogen fluoride due to its simple structure and longitudinal dipolar $S_0 \rightarrow S_1$ transition. The dipolar behaviour of this transition allows us to compare the coupling computed with the realistic molecule (Figure S2c) to the other cases where the molecule is approximated as a point-dipole (Figures S2a and S2b).

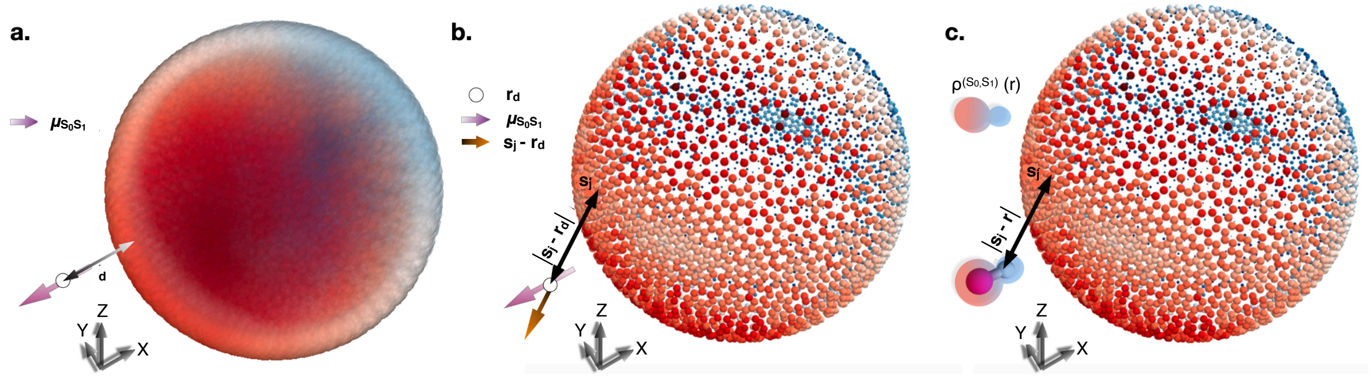

Figure S2: **Dipolar plasmonic mode of a spherical nanoparticle (diameter=10 nm) interacting with a molecule| a)** The molecule is represented as a point dipole distant ***d*** from the nanoparticle surface, while the nanoparticle is taken as a continuous Drude-Lorentz metal. The computed coupling is analytical, following ref.[17] **b)-c)** The plasmonic modes are computed at Q-PCM-NP diagonal level. In Panel **b)**, the molecule is represented as a point dipole corresponding to the $S_0 \rightarrow S_1$ transition dipole moment of the hydrogen fluoride molecule. The transition dipole moment $\mu_{S_0S_1} = 1.695$ Debye is computed at EOM-CCSD level. The EOM-CCSD calculations using the eT program[21] with a cc-pVTZ basis set. In Panel **c)** The molecule is represented with its $\rho^{(S_0,S_1)}(\boldsymbol{r})$ electronic transition density computed at CCSD level. The plasmon-molecule interactions are accounted via eq. S20 and eq. S29.

We use a 5 nm radius spherical nanoparticle, the same one shown in Figure S2b. To directly compare the results of our method with the analytical model of A. Delga *et al.*,[17] we define l as the angular momentum of the quantized spherical nanoparticle, recalling that the plasmonic modes are $2l + 1$ degenerate. We compute the analytical coupling values g_l^{an} by grouping and summing the degenerate plasmonic modes by angular momentum to remove potential artifacts due to the spatial orientation of the plasmonic modes. Following the derivation presented in ref.,[17] we recast the analytical coupling presented in eq. S30 into:

$$\left(g_l^{an,dip}\right)^2 = \frac{\sqrt{l}(l+1)^2\mu^2_{S_0,S_1}}{2}\sqrt{\frac{\Omega_P^2}{2l+1}\frac{r_s^{(2l+1)}}{d^{(2l+4)}}}, \tag{S53}$$

where l is the angular momentum associated with the nanoparticle plasmonic modes manifold (three dipolar modes, five quadrupolar modes and so on) and Ω_P is the plasma frequency appearing in Drude-Lorentz, which determines ω_p (eq. 7 of the main text). Here r_s and d are respectively the radius of the sphere and the distance of the point dipole from the nanoparticle surface.

To directly compare to the analytical ($g_l^{an,dip}$) value, we need to compute the interaction with the same modes with our method, hence taking into account the degeneracy of the sphere. To this aim, we sum the squared values of $g_p^{bem,dip}$ and $g_p^{bem,full}$ by angular momentum, respectively obtaining $(g_l^{bem,dip})^2$ and $(g_l^{full,dip})^2$. In Figure S3a, we compare the values of $(g_l)^2$ for the three models. As plasmonic modes for the test, we take the $l = 1$ (dipolar modes) and $l = 2$ (quadrupolar modes) of the sphere. We use EOM-CCSD for an accurate description of the dipolar molecule (hydrogen fluoride).

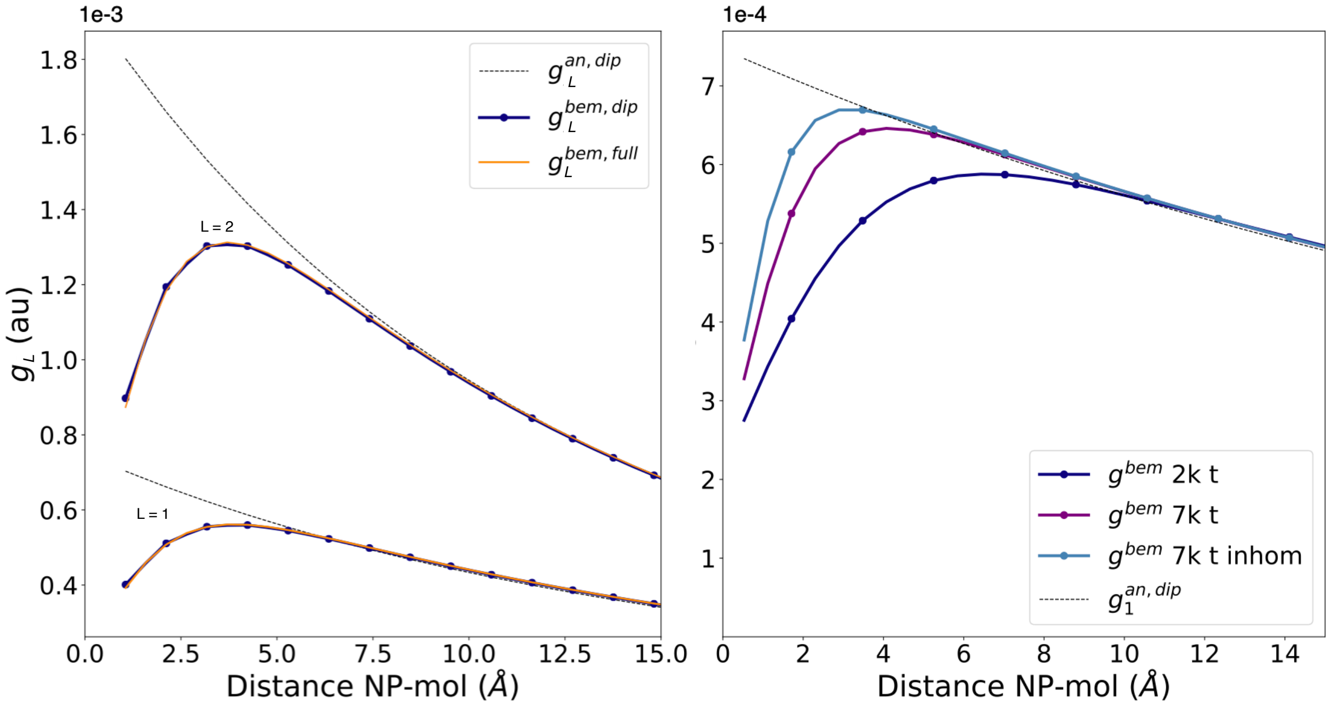

Figure S3: **Interaction between a molecule and the $\ell = 1$ (dipolar), $\ell = 2$ (quadrupolar) plasmonic modes of a nanosphere (diameter=10 nm)| a** Comparison between the analytical value reported in (dashed black line) with the $g_\ell^{bem,dip}$ and $g_\ell^{full,dip}$. **b** Interaction element for the $\ell = 1$ for the same case. The number of tesserae is increased from two thousands (labelled as 2k t, blue line) to seven thousands (labelled as 7k t, purple line). An even closer agreement between the analytical model and the BEM-based model is obtained by keeping seven thousands tesserae and making the mesh denser close to the molecule(labelled as 7k t inhom, light blue line).

A good agreement between the analytical model and the BEM variants is shown for both the interaction elements with $\ell = 1$ and $\ell = 2$ up to ~3 Å distance, comparable to the molecule-nanoparticle distance in plasmonic nanocavities and TERS setups.[8,22,23] For real molecules, at shorter distances the analytical model would not be realistic anyway, as further effects like charge transfer would take place. The same behaviour is shown for both the variants of the coupling computed with the BEM plasmonic modes. The choice of a small dipole-like molecule such as hydrogen fluoride ensures that the realistic molecule is fully comparable to the point-dipole case. Indeed, no difference is displayed when moving from point-dipole molecule ($g^{bem,dip}$) to the full molecular description ($g^{bem,full}$). The discrepancy between the analytical model and our method is then due to the numerical accuracy of the BEM calculation, that we can relate to density of the tesserae on the

nanoparticle surface. The effect becomes evident in Figure S3b, where we show the molecule-nanoparticle interaction for $\ell = 1$ obtained by varying the number and density of tesserae (and hence of the ASC). An increase in the quality of the description is obtained by increasing the number of tesserae from two thousands to seven thousands. A further improvement is observed by inhomogeneously distributing the seven thousand tesserae on the spherical surface, thickening the mesh close to the molecule. In the last case, the agreement between the analytical model and the BEM-based one holds down to ~ 2.5 Å, which is even smaller than the typical non-bond distances of 3.5 Å. The excellent agreement between our method and the analytical results ensures that the plasmon-molecule interaction in the near-field is reliably described with our method.

Computational Details

For the dimensions of the nanoparticles, please refer to Figure S1. The tassellation of the nanoparticles is performed with the GMSH[24] code by taking about 8k tesserae for the nanotip + support system, inhomogenoeusly distributed. We apply a linear-yet-steep gradient directed along the rotational axis of the nanotip and the support, thickening the tassellation towards the tip and on the top face of the support. By doing so, we obtain several hundreds of tesserae on the terminal part of the tip and 2k on the top face of the support. For the sphere instead different tassellations are compared in the last section of the present Supporting Information.

The calculation on the classical nanoparticles and the quantised modes with the Q-PCM-NP is currently implemented in the software TDPlas,[9] available at: https://github.com/stefano-corni/WaveT_TDPlas as a public repository. In our simulations for the nanoparticle + tip system we adopt the following Drude-Lorentz parameters:

- Ω_p^2=0.108 atomic units, corresponding to a bulk plasma frequency of 8.95 eV;
- γ=0.00077 atomic units (21 meV), corresponding to a decay rate of ;
- ω_0=0, no natural frequency meaning we are operating in the Drude limit.

We chose the Ω_p such that the frequency of the tip mode represented in Figure 1c would be positioned between the two quasi-degenerate transitions of the free-base porphyrin, namely at 4.06 eV. Such Ω_p is also very close to the Drude parameters for silver reported in ref.[25] The γ parameter is extracted from the same reference, always for the silver case.

The electronic wave functions and molecular properties are calculated using EOM-CCSD (cc-pVDZ basis set). The calculations on porphyrin and PNA are performed using equation-of-motion QED-CCSD-1 with an aug-cc-pVDZ basis set, except for the Jaynes-Cumming calculations (JC), which are performed using transition densities from EOM-CCSD. All calculations using QED-CC in this paper are performed using a local branch of the eT program.[21] The branch is expected to be released in the near future.

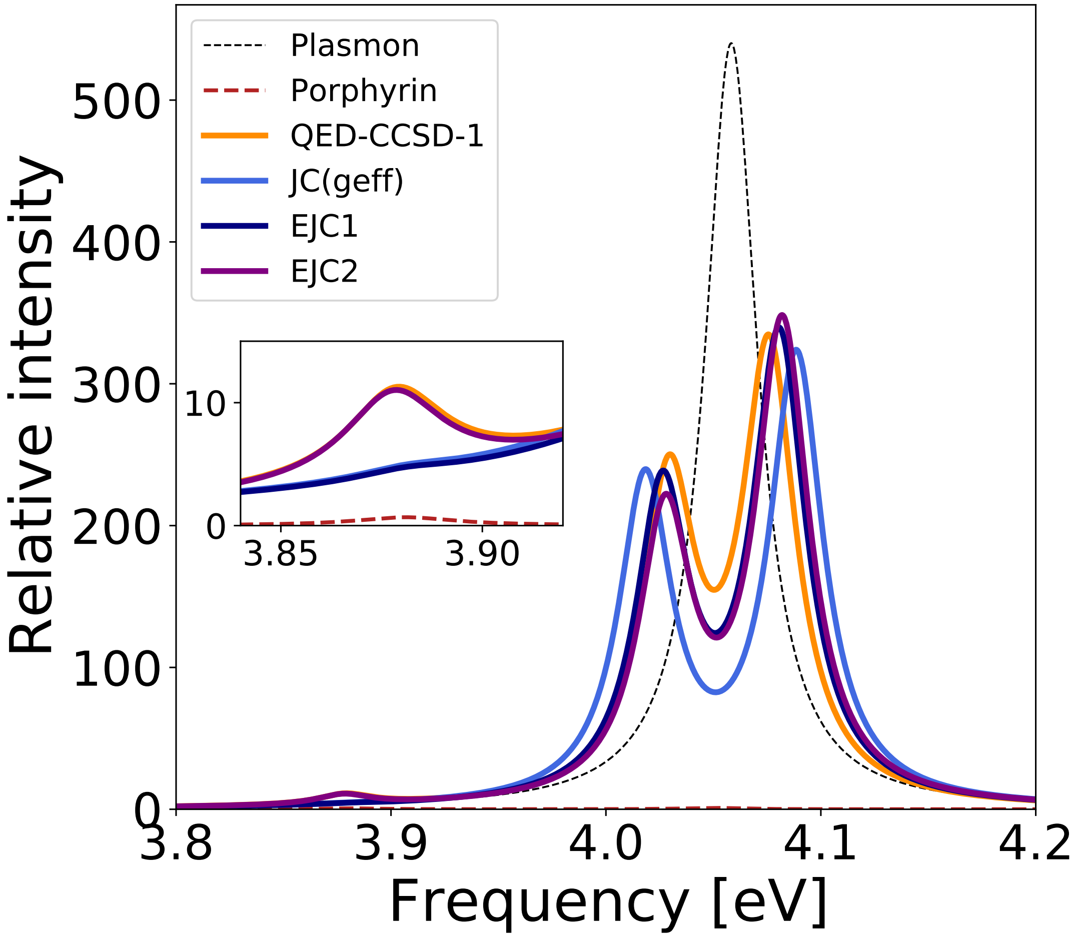

Figure S4: Porphyrin interacting with a plasmonic nanotip with transition energy ω = 4.06 eV (see Fig 2a). Oscillator strengths (intensity) are for porphyrin displaced 6 Å along x and 6 Å along y (see Fig 2b). Intensities are relative to porphyrin's strongest transition. The inset shows the same plot with only porphyrin. The Rabi splittings denoting JC are calculated using the two-state JC with g_{eff} and three-state (dark blue, EJC1 and purple, EJC2) Extended JC model with the excited state at 4.05 eV. The effective coupling for JC is $g_{\text{eff}} = \sqrt{g_1^2 + g_2^2}$. The EJC1 includes the ground state, the 4.05 eV and the 4.07 eV transition. EJC2 includes the ground state, the 3.88 eV and the 4.05 eV transitions. The comparison of the models in the region around 3.88 eV is displayed in the inset.

Many-state Jaynes-Cummings

The Jaynes-Cummings model used in the main text is defined by the Hamiltonian, $\hat{H} = \hat{H}_e + \hat{H}_p + \hat{g}_p$, in the basis of approximate eigenfunctions of $\hat{H}_e$ and eigenfunctions of $\hat{H}_p$ and applying the rotating-wave approximation. This leads to a 2-by-2 diagonalization problem to determine the dressed states and their energy,

$$\mathbf{H} = \begin{bmatrix} E_1 & g_{eff} \\ g_{eff} & E_2 \end{bmatrix}, \tag{S54}$$

where $g_{\text{eff}} = \sqrt{g_1^2 + g_2^2}$. When a three-state system is used for the molecule, then the dressed states are found by diagonalizing

$$\mathbf{H} = \begin{bmatrix} E_1 & g_1 & g_2 \\ g_1 & E_2 & 0 \\ g_2 & 0 & E_3 \end{bmatrix}. \tag{S55}$$

assuming that states 2 and 3 do not couple. In Figure S4 we show the different models applied to porphyrin interacting with a plasmonic nanotip. The effect of increasing the number of states has a small effect on the Rabi splitting and intensities. The three-state JC model, including the excited state with transition energy 3.88 eV, also reveals a new peak in the spectrum, already shown by QED-CCSD.

References

(1) Corni, S.; Tomasi, J. Enhanced response properties of a chromophore physisorbed on a metal particle. *J. Chem. Phys.* **2001**, *114*, 3739–3751.

(2) Corni, S.; Pipolo, S.; Cammi, R. Equation of Motion for the Solvent Polarization Apparent Charges in the Polarizable Continuum Model: Application to Real-Time TDDFT. *J. Phys. Chem. A* **2015**, *119*, 5405–5416.

(3) Cancès, E.; Mennucci, B.; Tomasi, J. A new integral equation formalism for the polarizable continuum model: Theoretical background and applications to isotropic and anisotropic dielectrics. *J. Chem. Phys.* **1997**, *107*, 3032–3041.

(4) Fuchs, R. Theory of the optical properties of ionic crystal cubes. *Phys. Rev. B* **1975**, *11*, 1732–1740.

(5) García de Abajo, F. J.; Aizpurua, J. Numerical simulation of electron energy loss near inhomogeneous dielectrics. *Phys. Rev. B* **1997**, *56*, 15873–15884.

(6) García de Abajo, F.; Howie, A. Retarded field calculation of electron energy loss in inhomogeneous dielectrics. *Phys. Rev. B* **2002**, *65*, 115418–17.

(7) Hohenester, U.; Trügler, A. MNPBEM – A Matlab toolbox for the simulation of plasmonic nanoparticles. *Comput. Phys. Commun.* **2012**, *183*, 370 – 381.

(8) Doppagne, B.; Neuman, T.; Soria-Martinez, R.; López, L. E. P.; Bulou, H.; Romeo, M.; Berciaud, S.; Scheurer, F.; Aizpurua, J.; Schull, G. Single-molecule tautomerization tracking through space- and time-resolved fluorescence spectroscopy. *Nat. Nanotechnol.* **2020**, *15*, 207–211.

(9) Pipolo, S.; Corni, S. Real-Time Description of the Electronic Dynamics for a Molecule Close to a Plasmonic Nanoparticle. *J. Phys. Chem. C* **2016**, *120*, 28774–28781.

(10) Dall'Osto, G.; Gil, G.; Pipolo, S.; Corni, S. Real-time dynamics of plasmonic resonances in nanoparticles described by a boundary element method with generic dielectric function. *J. Chem. Phys.* **2020**, *153*, 184114.

(11) Neuman, T.; Esteban, R.; Casanova, D.; García-Vidal, F. J.; Aizpurua, J. Coupling of Molecular Emitters and Plasmonic Cavities beyond the Point-Dipole Approximation. *Nano Lett.* **2018**, *18*, 2358–2364.

(12) Corni, S.; Tomasi, J. Theoretical evaluation of Raman spectra and enhancement factors for a molecule adsorbed on a complex-shaped metal particle. *Chem. Phys. Lett.* **2001**, *342*, 135–140.

(13) Jaynes, E. T.; Cummings, F. W. Comparison of quantum and semiclassical radiation theories with application to the beam maser. *Proc. IEEE* **1963**, *51*, 89.

(14) Galego, J.; Garcia-Vidal, F. J.; Feist, J. Cavity-Induced Modifications of Molecular Structure in the Strong-Coupling Regime. *Phys. Rev. X* **2015**, *5*, 041022.

(15) Kowalewski, M.; Bennett, K.; Mukamel, S. Non-adiabatic dynamics of molecules in optical cavities. *J. Chem. Phys.* **2016**, *144*, 054309.

(16) Herrera, F.; Spano, F. C. Absorption and photoluminescence in organic cavity QED. *Phys. Rev. A* **2017**, *95*, 053867.

(17) Delga, A.; Feist, J.; Bravo-Abad, J.; Garcia-Vidal, F. J. Quantum Emitters Near a Metal Nanoparticle: Strong Coupling and Quenching. *Phys. Rev. Lett.* **2014**, *112*, 253601.

(18) Haugland, T. S.; Ronca, E.; Kjønstad, E. F.; Rubio, A.; Koch, H. Coupled Cluster Theory for Molecular Polaritons: Changing Ground and Excited States. *Phys. Rev. X* **2020**, *10*, 041043.

(19) Tomasi, J.; Mennucci, B.; Cammi, R. Quantum Mechanical Continuum Solvation Models. *Chem. Rev.* **2005**, *105*, 2999–3094.

(20) Helgaker, T.; Jørgensen, P.; Olsen, J. *Molecular Electronic-Structure Theory*; John Wiley & Sons, Ltd: Chichester, UK, 2000.

(21) Folkestad, S. D. et al. eT 1.0: an open source electronic structure program with emphasis on coupled

cluster and multilevel methods. 2020.
(22) Chikkaraddy, R.; de Nijs, B.; Benz, F.; Barrow, S. J.; Scherman, O. A.; Rosta, E.; Demetriadou, A.; Fox, P.; Hess, O.; Baumberg, J. J. Single-molecule strong coupling at room temperature in plasmonic nanocavities. *Nature* **2016**, *535*, 127.
(23) Hugall, J. T.; Singh, A.; van Hulst, N. F. Plasmonic Cavity Coupling. *ACS Photonics* **2018**, *5*, 43–53.
(24) Geuzaine, C.; Remacle, J.-F. Gmsh: A 3-D finite element mesh generator with built-in pre- and post-processing facilities. *Int. J. Numer. Methods Eng.* **2009**, *79*, 1309–1331.
(25) Zeman, E. J.; Schatz, G. C. An accurate electromagnetic theory study of surface enhancement factors for silver, gold, copper, lithium, sodium, aluminum, gallium, indium, zinc, and cadmium. *J. Phys. Chem.* **1987**, *91*, 634–643.
